# Spin-dependent vibronic response of a carbon radical ion in two-dimensional WS_2_

**DOI:** 10.1038/s41467-021-27585-x

**Published:** 2021-12-15

**Authors:** Katherine A. Cochrane, Jun-Ho Lee, Christoph Kastl, Jonah B. Haber, Tianyi Zhang, Azimkhan Kozhakhmetov, Joshua A. Robinson, Mauricio Terrones, Jascha Repp, Jeffrey B. Neaton, Alexander Weber-Bargioni, Bruno Schuler

**Affiliations:** 1 Molecular Foundry, Lawrence Berkeley National Laboratory, Berkeley, CA 94720 USA; 2 Materials Sciences Division, Lawrence Berkeley National Laboratory, Berkeley, CA 94720 USA; 3 Department of Physics, University of California at Berkeley, Berkeley, CA 94720 USA; 4 Walter-Schottky-Institut and Physik-Department, Technical University of Munich, Garching, 85748 Germany; 5 Department of Materials Science and Engineering, The Pennsylvania State University, University Park, PA 16082 USA; 6 Center for Two-Dimensional and Layered Materials, The Pennsylvania State University, University Park, PA 16802 USA; 7 Department of Physics and Department of Chemistry, The Pennsylvania State University, University Park, PA 16802 USA; 8 Institute of Experimental and Applied Physics, University of Regensburg, Regensburg, 93040 Germany; 9 Kavli Energy Nanosciences Institute at Berkeley, Berkeley, CA 94720 USA; 10 nanotech@surfaces Laboratory, Empa—Swiss Federal Laboratories for Materials Science and Technology, Dübendorf, 8600 Switzerland

**Keywords:** Electronic properties and materials, Two-dimensional materials, Two-dimensional materials

## Abstract

Atomic spin centers in 2D materials are a highly anticipated building block for quantum technologies. Here, we demonstrate the creation of an effective spin-1/2 system *via* the atomically controlled generation of magnetic carbon radical ions (CRIs) in synthetic two-dimensional transition metal dichalcogenides. Hydrogenated carbon impurities located at chalcogen sites introduced by chemical doping are activated with atomic precision by hydrogen depassivation using a scanning probe tip. In its anionic state, the carbon impurity is computed to have a magnetic moment of 1 *μ*_B_ resulting from an unpaired electron populating a spin-polarized in-gap orbital. We show that the CRI defect states couple to a small number of local vibrational modes. The vibronic coupling strength critically depends on the spin state and differs for monolayer and bilayer WS_2_. The carbon radical ion is a surface-bound atomic defect that can be selectively introduced, features a well-understood vibronic spectrum, and is charge state controlled.

## Abstract (web summary)

Spin-polarized defects in 2D materials are attracting attention for future quantum technology applications, but their controlled fabrication is still challenging. Here, the authors report the creation and characterization of effective spin 1/2 defects via the atomically-precise generation of magnetic carbon radical ions in 2D WS_2_.

## Introduction

For decades, defects in semiconductors and insulators have been widely utilized to control the electronic, optical, and catalytic properties of solids. More recently, the electron spin associated with atomic defects in crystals has attracted enormous interest in light of their potential applications in quantum technologies^1–3^. The strong confinement of atomic-scale quantum systems imposes a large characteristic energy range that is amenable for pushing quantum technologies beyond cryogenic environments^4^, while the limited system size promotes favorable coherence properties^4^.

However, generating identical defects with the necessary atomic precision, designing them to be tunable by external fields, and often even knowing their exact identities have remained unsolved challenges in the field. In this respect, two-dimensional (2D) materials offer new opportunities for the atomically-precise generation and control of defect spins^5,6^. There have been extensive efforts to utilize boron nitride or transition metal dichalcogenides (TMDs) as a platform for so-called quantum emitters, that is spin-carrying defects that can be optically detected. In boron nitride, quantum emission has been attributed to carbon impurities^7^ among other defects^8–10^. In TMDs similar behavior has been reported but is mostly ascribed to mesoscopic strain profiles^11^. Atomic defects in TMDs have been generated previously by transmission electron microscopy^12^, and ion beam lithography^13,14^. However, the generated defects are often not identical and have mostly non-spin-polarized states.

Here, we introduce the carbon radical ion (CRI) in tungsten disulfide (WS_2_) as an effective spin-1/2 system that can be created with atomic precision while keeping the surrounding atomic structure virtually unchanged. We precisely characterize the coupling of the spin-polarized local defect states generated by the CRI with its host lattice. Inherent to any solid-state atomic quantum system, the defect-specific electron-phonon interaction is often a dominant decay and decoherence channel^15^. Here, we demonstrate that the electron-phonon coupling associated with the two discrete electronic CRI defect states is limited to a only few vibrational modes and exhibits a distinct spin and layer dependence.

Carbon impurity defects at chalcogen sites (C_X_, X = S, Se) are created by scanning tunneling microscopy (STM)-induced hydrogen desorption from carbon–hydrogen (CH) complexes. Such CH impurities are frequently found in synthetic WS_2_ and WSe_2_^16^ but can also be deliberately created by post-synthetic methane plasma treatment, as shown here for WS_2_^16,17^. We induce the hydrogen depassivation of CH_X_ by a voltage pulse from the STM tip, which is highly reproducible and atomically precise. For WS_2_, the Fermi level alignment with the graphene substrate results in a negatively charged carbon impurity with a radical character that we refer to as a CRI and denote as C${}_{\,{{\mbox{S}}}\,}^{\bullet -}$. The CRI has an occupied spin-polarized defect state with a net magnetic moment of 1 μ_B_, as predicted by our density functional theory (DFT) calculations. In WSe_2_, we find C_Se_ is neutral and thus has no magnetic moment. Moreover, we quantify the vibronic coupling of a single CRI to the host lattice by inelastic transport spectroscopy and using DFT calculations. We find that the CRI defect orbitals couple predominantly to just a few vibrational modes. However, the coupling strength critically depends on the constituent state of the CRI two-level system as well as on the number of TMD layers.

## Results and discussion

**Fig. 1:**
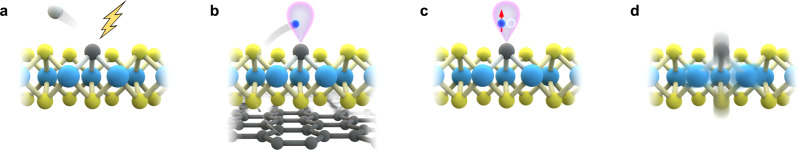
Creation and main attributes of the carbon radical ion (CRI). **a** Schematic illustration of the hydrogen depassivation of the CH impurity. **b** Electron transfer from the substrate into the dangling C_S_ bond. **c** Magnetic moment of the carbon radical ion (CRI). **d** CRI vibronic coupling to the TMD host lattice. Color code: H (white), C (gray), S (yellow), W (blue), electron (small blue) with spin (red arrow).

In the following, we discuss our three primary conclusions: the hydrogen depassivation of the CH impurity and formation of the CRI, the two spin-polarized defect states associated with the CRI, and the vibronic coupling of the CRI defect states with the TMD host lattice (see Fig. 1).

### Hydrogen depassivation of a CH impurity

**Fig. 2:**
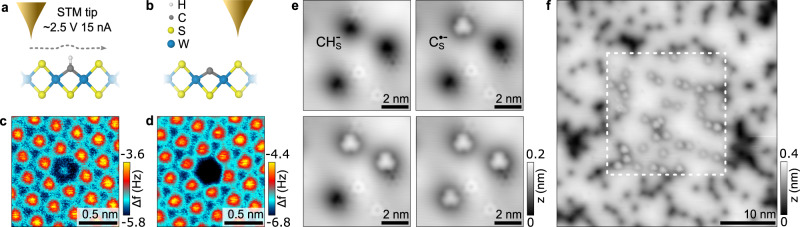
Hydrogen desorption from CH_S_ impurities in deliberately CH-doped WS_2_. **a**, **b** Atomic structure of a carbon-hydrogen complex (CH_S_) and a carbon (C_S_) substituent at a sulfur site in WS_2_. The pathway of the tip for the dehydrogenation process is shown in (**a**). **c**, **d** Constant height CO-tip nc-AFM images of the CH_S_ (**c**) and C_S_ (**d**) defect. **e** Sequential, controlled conversion of CH${}_{\,{{\mbox{S}}}\,}^{-}$ to C${}_{\,{{\mbox{S}}}\,}^{\bullet -}$ by H desorption (*V* = 1.1 V, *I* = 100 pA). The sample bias and current were increased to 2.5 V and 15 nA and rastered over an individual defect with images taken between each conversion. **f** Large scale image showing conversion of a center subsection (20 × 20 nm^2^) outlined by the white dashed box (*V* = 1.2 V, *I* = 100 pA).

In Fig. 2, STM and CO-tip noncontact atomic force microscopy (nc-AFM) images of CH${}_{\,{{\mbox{S}}}\,}^{-}$ defects in deliberately CH-doped monolayer WS_2_ (0.6% atomic doping concentration) are presented. In the nc-AFM images, CH${}_{\,{{\mbox{S}}}\,}^{-}$ defects appear as a small protrusion at a sulfur site (Fig. 2c), in excellent agreement with the simulated AFM contrast obtained from the relaxed geometry using DFT calculations (Supplementary Fig. 20). In STM, CH${}_{\,{{\mbox{S}}}\,}^{-}$ is imaged as a large, circular depression at positive bias resulting from upwards band bending due to the negative charge^18,19^. After scanning the tip over the defect at high applied sample biases and high tunneling current set-points (~2.5 V and ~15 nA), a dramatic change in the STM and AFM contrast is observed. In AFM, the small protrusion disappears (see Fig. 2d). In STM, a three-fold symmetric, bright orbital structure on a dark background is observed at positive voltage. Based on the prior knowledge of the precursor defect by targeted doping, the nc-AFM simulations, and the characteristic electronic fingerprint of the converted defect (discussed below), we show that the conversion process is controlled desorption of the hydrogen atom from the CH complex. The hydrogen desorption by the STM tip is likely a resonant process where tunneling into an unoccupied CH_S_ defect state weakens the C–H bond. This defect state as identified by DFT calculations exhibits a local anti-bonding character with a nodal plane between the carbon and hydrogen atom, supporting this hypothesis^16^. While the process is stochastic in nature, we find a minimum value of 2.3 V, required for the C–H dissociation. Tunneling at negative bias with a comparable magnitude does not result in H-desorption.

Hydrogen desorption by STM has been reported for hydrogen-terminated silicon surfaces^20–22^ and organic molecules^23,24^. As with these systems, removal of the hydrogen creates a dangling bond with a radical character, hence the defect becomes “depassivated”. The dehydrogenation of the CH defect is very reliable (success rate of about 95%), and can be performed with single-atom precision as seen in Fig. 2e. Larger patterns can be written by scanning the surface at elevated bias and currents, shown in Fig. 2f. Occasionally, a hydrogen atom reattaches from a previously depassivated C_S_ defect while scanning at high bias (see Supplementary Fig. 3). We speculate that an H atom residing close to the tip apex transfers back and passivates the defect again, as previously suggested^25^. This shows that the process is reversible. Even more rarely, the entire CH complex is removed irreversibly, creating a sulfur top vacancy.

Hydrogen depassivation of CH-doped TMDs can generate single defects in a 2D material with atomic precision. This is a sought-after capability for defect-based quantum systems not yet demonstrated for a 2D material. In analogy to the single transistor technologies based on hydrogen resist lithography in silicon^21,26^, we also envision that the dangling bonds of C_X_ could be used as a predefined reactive docking site for other atoms or molecules. This approach will enable embedding functional atoms in a 2D manifold in a spatially controlled manner.

### Electronic and magnetic properties of the CRI

**Fig. 3:**
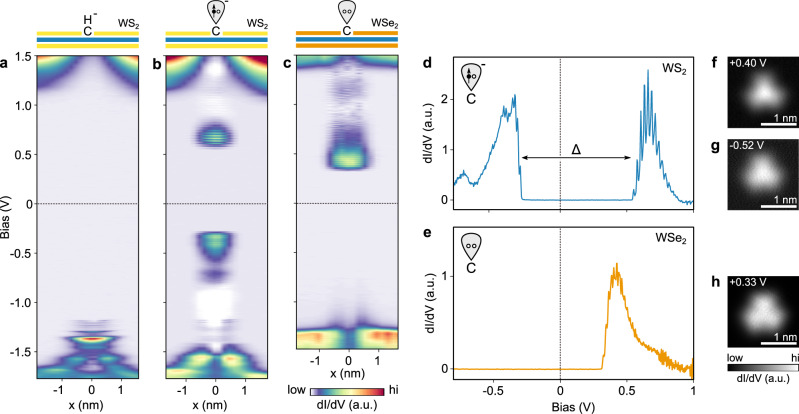
Tunneling spectroscopy of a carbon impurity in WS_2_ and WSe_2_. **a**, **b** Constant height dI/dV measurement across CH${}_{\,{{\mbox{S}}}\,}^{-}$ in monolayer WS_2_ before (**a**) and after (**b**) H dissociation. Both CH${}_{\,{{\mbox{S}}}\,}^{-}$ and C${}_{\,{{\mbox{S}}}\,}^{\bullet -}$ are negatively charged. The half-occupied dangling bond state of the carbon radial ion appears as two resonances in the band gap at positive and negative bias. **c** Constant height dI/dV measurement across C${}_{\,{{\mbox{Se}}}\,}^{0}$ in monolayer WSe_2_. In contrast to WS_2_, the carbon dangling bond in WSe_2_ is unoccupied and the defect is neutral. **d**, **e** d*I*/d*V* spectrum recorded at the center of the C${}_{\,{{\mbox{S}}}\,}^{\bullet -}$ and C${}_{\,{{\mbox{Se}}}\,}^{0}$ defect, respectively. The energy difference Δ = 705 meV between electron de- and attachment in (**d**) results from a combination of Coulomb repulsion and spin-splitting. **f**, **g** Constant-height dI/dV maps of the C${}_{\,{{\mbox{S}}}\,}^{\bullet -}$ resonance at positive and negative bias. Their similar orbital shape indicates that they originate from the same half-occupied defect state. **h** Constant-height d*I*/d*V* map of the single C_Se_ resonance.

The drastic change in STM contrast after dehydrogenation suggests a significant reconfiguration in the defect electronic structure. Scanning tunneling spectra across the CH${}_{\,{{\mbox{S}}}\,}^{-}$ defect in monolayer WS_2_ are shown in Fig. 3a. The negative charge localized at the defect gives rise to a strong upwards band bending, explaining why it appears as a dark extended depression in STM images at positive bias voltage. At negative sample bias, multiple defect states are observed, which we attribute to hydrogenic bound and resonant states of the screened Coulomb potential, as we reported recently^18^.

After hydrogen desorption, two prominent defect states emerge deep in the WS_2_ bandgap, one at positive (~0.6 V) and the other at negative (~−0.3 V) sample bias, as seen in Fig. 3b, d. These highly localized defect states are well decoupled from the dispersive bulk WS_2_ band structure. Each state exhibits an oscillatory fine structure that is a signature of the vibronic coupling to the TMD lattice, which will be discussed later in detail. Spatial imaging of these defect states (Fig. 3f, g) reveals that they have threefold symmetry and nearly identical orbital shapes, strongly suggesting that the two resonances originate from a single open-shell defect state^27^. The gap between the defect states is slightly larger on bilayer (Δ = 770 meV) than on monolayer (Δ = 705 meV) WS_2_. Note that these numbers were corrected for the ~11% voltage drop^28^ across the TMD—Gr/SiC interface in our double-barrier tunneling junction geometry. The same upwards band bending before and after dehydrogenation indicates that the defect is still negatively charged, consistent with the persisting dark halo in STM images of C_S_.

**Fig. 4:**
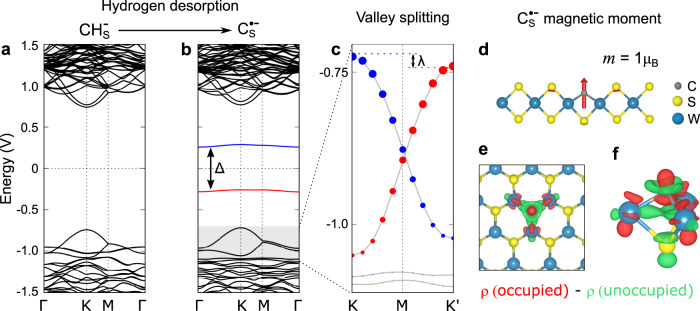
Calculated electronic and magnetic structure of a CRI. **a** Calculated band structure of the negatively charged CH_S_ impurity using DFT-PBE in a 6 × 6 supercell with SOC. Note that in free-standing WS_2_ the defect would be charge neutral and the valence band half-filled. Zero energy is set to the middle of the energy gap for comparison. **b** Calculated band structure of C${}_{\,{{\mbox{S}}}\,}^{\bullet -}$. C_S_ on free-standing WS_2_ features only a single unoccupied defect state in the center of the WS_2_ bandgap. Upon charging from the substrate, the now half-filled defect state is stabilized by spin-splitting (Δ = 593 meV, an underestimate due to the use of DFT-PBE). The gray box marks the energy range displayed in (**c**). **c** The magnetic moment of the unpaired defect spin lifts the energy degeneracy of the *K* and *K*' valley (*λ* = 16 meV at a defect density of 4.7 × 10^13^ cm^−2^). Red and blue colors in (**b**) and (**c**) indicate opposite spin polarization. Blue (red) dots in (**c**) represent contribution of W *l* = 2, *m*_*j*_ = −5/2(+5/2) state. **d** Side view of the defect-localized magnetic moment of *m* = 1 μ_B_ in the out-of-plane spin configuration (for moments > 0.01 μ_B_). **e**, **f** Isosurface (±0.0008 *e*/Å) of the difference in electron density. The occupied defect state has a higher charge density in the vicinity of the carbon atom (red). The unoccupied defect state has a higher charge density in the W plane (green).

In WSe_2_ on the other hand, the carbon defect becomes charge-neutral after dehydrogenation (CH${}_{\,{{\mbox{Se}}}\,}^{-}\,$ → C${}_{\,{{\mbox{Se}}}\,}^{0}$). The charge neutrality can be deduced from the absence of band bending thus leading to a disappearance of the dark halo around the defect when imaged at positive sample biases upon dehydrogenation (see Supplementary Fig. 8), and the disappearance of associated hydrogenic states as seen in Fig. 3c. Moreover, the carbon impurity exhibits only a single, fully unoccupied defect state in the WSe_2_ bandgap, and no state at negative bias is observed (Fig. 3e). This neutral charge is a consequence of the different band alignments of WS_2_ and WSe_2_ with the Gr/SiC substrate. In the WSe_2_/Gr/SiC heterostructure, the Fermi level lies roughly in the center of the WSe_2_ bandgap, whereas it is 443 meV higher for WS_2_^29^. Accordingly, the underlying substrate does not donate an electron to the dangling bond-like defect state of C_Se_ in WSe_2_ and thus remains empty. Alternatively, the charge state of a C_X_ could be controlled by changing the graphene Fermi level electrostatically.^30,31^ Prior DFT calculations of a neutral carbon impurity in WS_2_ featured a single unoccupied defect state in the energy gap^17^. Addition of one electron to form a negatively charged carbon impurity in WS_2_ results in a spin polarization and exchange-induced splitting of the in-gap state, as shown in Fig. 4b. The total energy gain of the spin-polarized ground state as compared to the less favorable non-magnetic configuration is 142 meV per single carbon atom (see Supplementary Fig. 16 for details). Hence, the negatively charged carbon impurity is characterized by a spin-split two-level system of which the lower level is occupied by one electron. Spatial maps of these defect states computed from DFT show they are strongly localized and almost identical to each other, in agreement with experimental observations (Supplementary Fig. 9). Orbital projected density of states from our DFT calculations reveals that the defect states are strongly hybridized and possess C 2*p*, W 5*d*, and S 3*p* orbitals character. The charged carbon impurity is computed to possess a magnetic moment of 1 μ_B_ from our DFT calculations, with a spin distribution closely localized at the carbon atom as shown in Fig. 4d. Based on these calculations, we conclude that the defect can be described as a CRI C${}_{\,{{\mbox{S}}}\,}^{\bullet -}$, or CRI; and we assign the two experimentally observed in-gap states as the occupied and unoccupied spin-split defect states associated primarily with the carbon anion.

While the two defect states closely resemble a spin-1/2 system, the hybrid character of the states, with contributions from mainly four atoms, in conjunction with SOC, leads to a small but finite magnetocrystalline anisotropy, as reported before in other solid-state spin systems^32^. Our DFT calculations predict magnetocrystalline anisotropy energy (MAE) of 0.1 meV with an easy-axis perpendicular to the WS_2_ plane. Because the crystal field breaks the rotational symmetry of the impurity, the total angular momentum *J* is strictly speaking not a good quantum number. Rather it is more appropriate to classify defect states by the irreducible representation of the symmetry group associated with the defect site, in this case, the *C*_3*v*_ point group. Our DFT calculations indicate that the spatial part of both the occupied and unoccupied defect states transform symmetrically under the *C*_3*v*_ point group and can be labeled by the *A*_1_ irreducible representation. Since the dimension of *A*_1_ is one, the two defect states are non-degenerate. The Mo_S_ antisite defect in MoS_2_ with the same symmetry has been recently predicted to feature a giant MAE of about 500 meV^33^. The comparably small MAE of C${}_{\,{{\mbox{S}}}\,}^{\bullet -}$ might be expected given the small spin–orbit coupling (SOC) of carbon, similar to other light element color centers.

At ultra-low temperatures (where MAE ≫ k_B_*T*), the defect magnetic moment will be fixed in space. At a finite defect density, a net magnetic order could induce a spin-dependent shift of the spin-polarized WS_2_ valence band electrons as shown in Fig. 4c, lifting the energy degeneracy between the *K* and *K*’ valleys due to time-reversal symmetry breaking. This energy shift *λ* is calculated to be 16 meV for an ordered array of ferromagnetically coupled defects with a density of 4.7 × 10^13^ cm^−2^ (5 × 5 unit cell) with the PBE functional^34^ (see Supplementary Fig. 19 for density dependence).

The unpaired electron of the negatively charged CRI constitutes an effective spin-1/2 system. In organic chemistry, CRIs have been studied for decades in the context of reaction intermediates. Unpaired spins of organic compounds can be detected by electron spin resonance (ESR)^35^. Owing to their high reactivity, free radicals are usually very short-lived. In our experiments, the UHV conditions stabilize the CRIs, but alternatively, an inert capping layer such as hBN could be employed to protect the carbon dangling bond. It is also worth noting that isotopically pure CRIs can be easily prepared by using commercially available ^12^C or ^13^C clean variants of methane in the plasma treatment. Moreover, the low abundance of non-zero nuclear spin isotopes in certain TMDs and the intrinsically reduced spin densities in low-dimensional materials makes the TMD matrix a great host for defect spins^36^. While spin-bath fluctuations can be expected to act favorably in 2D TMDs, electron–phonon coupling could pose another significant source of spin decoherence, which will be discussed next.

### Vibronic coupling of the CRI

Each C_S_ defect resonance above and below the Fermi energy features characteristic, equidistant peaks, a consequence of a strong electron-phonon interaction which can be probed by the transient attachment of an electron (at positive bias voltage) or a hole (at negative bias voltage) associated with the tunneling process. Understanding the vibronic coupling of solid-state atomic quantum systems is critical as it can limit the attainable coherence times^15^. However, phonon sidebands can be effectively suppressed by the frequency-selective emission enhancement of a resonant cavity^15,37^. Moreover, low-loss local vibrations or surface acoustic phonons are analogous to nanomechanical resonators that can be used to store or coherently transmit quantum information between remote quantum systems^38–40^. Electron–phonon coupling is particularly relevant for very localized states, which in general lead to larger lattice relaxations. This applies to any deep center in wide-bandgap semiconductors. The theoretical framework of electron–phonon interactions at bulk defects was developed by Huang and Rhys, as well as Pekar in the 1950s and 1960s^41^. More recently, ab initio calculations of vibronic coupling to defect states in diamond ^42^ and in 2D materials have been a subject of active research^43,44^.

In scanning tunneling spectroscopy (STS) measurements, inelastic scattering between localized charged excitations and vibrational modes is well known to lead to characteristic sideband structures. Such phenomena have been observed for molecules on surfaces^45–47^, color centers in dielectrics^48^, and semiconductor quantum well tunneling devices^49^, where particular vibrational modes have been found to couple to localized electronic states. As mentioned in the previous section, when a CRI is introduced, we are able to clearly resolve this sideband structure in our d*I*/d*V* measurements (Fig. 3d). Interestingly this sideband structure differs substantially depending on the spin-state of the CRI, with the electron attachment (positive bias) exhibiting a clean Franck–Condon-like vibronic profile while the hole attachment (negative bias) exhibits a more complex fine structure, possibly involving multiple phonons. That the vibronic structure of the spin-split defect state is sensitive to the spin state is somewhat unintuitive given that these states derive from the same non-spin-split parent state and have essentially the same orbital structure. We note that the interaction between the spin-split localized defect state and bath of harmonic phonons can be described by an effective independent spin-boson Hamiltonian^50,51^, as detailed in the Methods. From the exact solution of this model Hamiltonian, we can derive the electron spectral function *A*_*σ*_(*ω*), that is

1 $${A}_{\sigma }(\omega )=2\pi \mathop{\sum }\limits_{\{{l}_{1}{l}_{2}...{l}_{n}\}}^{\infty }\left[\left(\mathop{\prod }\limits_{\nu =1}^{n}{e}^{-{S}_{\nu \sigma }}\frac{{S}_{\nu \sigma }^{{l}_{\nu }}}{{l}_{\nu }!}\right)\delta \left(\hslash \omega -{\bar{\epsilon }}_{\sigma }-\mathop{\sum }\limits_{\mu =1}^{n}\hslash {\omega }_{\mu }{l}_{\mu }\right)\right],$$

where *ω*_*ν*_ is the frequency of the vibrational mode *ν*, and ${S}_{\nu \sigma }={({g}_{\nu \sigma }/{\omega }_{\nu })}^{2}$ is the Huang–Rhys factor, related in turn to the defect state-phonon coupling strength *g*_*νσ*_; and *l*_*ν*_ is an integer. Note that the Huang–Rhys factors and defect-phonon coupling strengths have a spin index, *σ*, while the vibrational frequencies do not, reflecting the fact that the frequencies are insensitive to the spin states while the coupling, in general, is not. Finally, ${\bar{\epsilon }}_{\sigma }$ denotes the electronic defect state energy, including the vibrational self-energy.

We calculate the vibrational frequencies, *ω*_*ν*_, using density functional perturbation theory (DFPT). Subsequently, the spin-dependent Huang–Rhys factors, *S*_*νσ*_, are extracted from a spin-polarized finite difference DFT calculation of the electron-phonon coupling, using eigendisplacements from DFPT. In this manner, all quantities appearing in Eq. (5) are determined from the first principles with no adjustable parameters (for details see Methods).

**Fig. 5:**
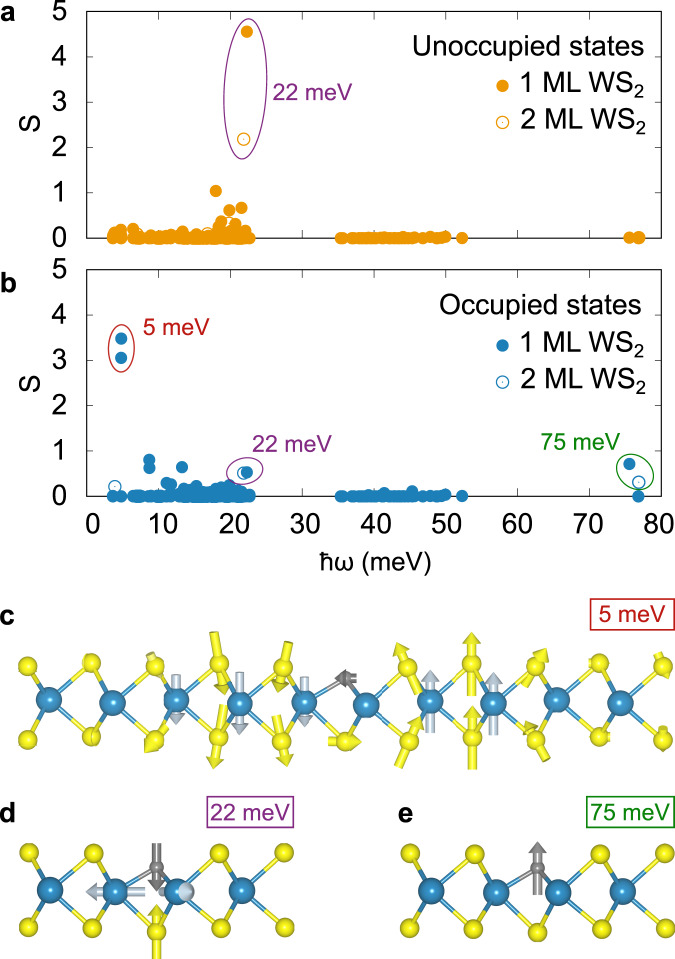
Calculated electron–phonon coupling. **a**, **b** Calculated Huang–Rhys factors *S* for unoccupied (**a**) and occupied (**b**) defect state for different phonons of frequency ℏ*ω*. Filled (open) circles represent 1ML (2ML). The values are calculated at the Γ point in 5 × 5, 6 × 6, and 7 × 7 supercells (shown all together). The Γ point of these different unit cells effectively samples TMD modes with different wavevectors, which leads to convergence challenges for the low-energy resonant modes involving the defect at 5 meV. The two local modes at 22 and 75 meV are converged for supercells larger than 5 × 5. **c**–**e** Side projections of (**c**) the resonant mode at 5 meV (flexural mode, red circle in (**b**)) and the dominant local vibrational modes at 22 meV (breathing mode, purple circles in (**a**) and (**b**)) (**d**) and 75 meV (carbon out-of-plane oscillation, green circle in (**b**)) (**e**). The experimental lattice parameter is used for all calculations. For 1ML (2ML), PBE (LDA) exchange-correlation functional is used.

In our DFT calculations of a negatively charged carbon impurity, we find two vibrational modes spatially localized around the defect that exhibit significant coupling to the defect state, with frequencies *ℏω* = 22 and 75 meV. The 22 meV mode is located 0.3 meV below the top of the acoustic branch of the pristine WS_2_ monolayer and corresponds to an out-of-phase breathing motion involving the C–S bond and the neighboring three W atoms (Fig. 5d). Interestingly, the defect state-phonon coupling associated with this mode is very sensitive to spin and occupation of the CRI state: while we compute *S* = 4.5 for the unoccupied CRI state, we obtain *S* = 0.5 for the occupied state. This nearly order of magnitude difference can be attributed to the spin-dependence of the change in XC potential in response to specific phonon perturbations and distinct spatial parts of the wavefunctions of the two spin-polarized defect states.

To illustrate the origin of this spin-dependent coupling, we partition the Kohn-Sham Hamiltonian into two terms, a kinetic + ionic + Hartree term (*T* + *V*_ext_ + *V*_H_) and an XC term (${V}_{\,{{\mbox{xc}}}\,}^{\sigma }$), and express the total defect-state electron-phonon coupling as a sum of the two contributions, namely

2 $${g}_{\nu \sigma }=\left\langle {\psi }_{\sigma }\left|{\partial }_{\nu }(T+{V}_{{{{{{{{\rm{ext}}}}}}}}}+{V}_{H})\right|{\psi }_{\sigma }\right\rangle +\left\langle {\psi }_{\sigma }\left|{\partial }_{\nu }{V}_{\,{{\mbox{xc}}}\,}^{\sigma }\right|{\psi }_{\sigma }\right\rangle ,$$

where *ψ*_*σ*_ labels the defect state of interest and ∂_*ν*_ denotes a derivative with respect to the amplitude of displacements of phonon mode *ν*. The utility of the above decomposition is that it isolates the spin-dependent part of the KS Hamiltonian ${V}_{{{{{{{{\rm{xc}}}}}}}}}^{\sigma }$. In Supplementary Fig. 13, we explicitly show how the kinetic + ionic + Hartree (KIH) term and exchange-correlation (XC) term contribute to the overall defect state-phonon coupling. For the 75 meV mode, we find for the occupied state the KIH and XC contributions add constructively while for the unoccupied state they approximately cancel out, leading to significant differences in vibrational coupling for the two states. In contrast, for the 22 meV mode, contributions from ${V}_{\,{{\mbox{xc}}}\,}^{\sigma }$ dominate the coupling strength for the unoccupied state while it is marginal for the occupied state.

Separately, in Supplementary Fig. 13, we show the KIH term also exhibits some spin-dependence, associated with the different character of the spin-split defect state wavefunction *ψ*_*σ*_. To further explore this point, we plot in Fig. 4e, f the difference of the defect state electron densities, Δ*ρ* = *ρ*_*o*_ − *ρ*_*u*_, where *ρ*_*o*/*u*_ = ∣*ψ*_*o*/*u*_∣^2^, clearly revealing the difference of the two defect states. The occupied defect state features more charge density in the vicinity of the carbon atom, which increases the coupling to the 75 meV mode, corresponding to a local out-of-plane C vibration (Fig. 5e). The unoccupied defect state has more charge density in the W plane, which leads to stronger coupling to the 22 meV mode (see Fig. 4e, f). We find that the vibronic coupling to a defect state becomes significant as the degree of the localization of the electronic wave function is sizable at the lattice sites where the vibration occurs.

**Fig. 6:**
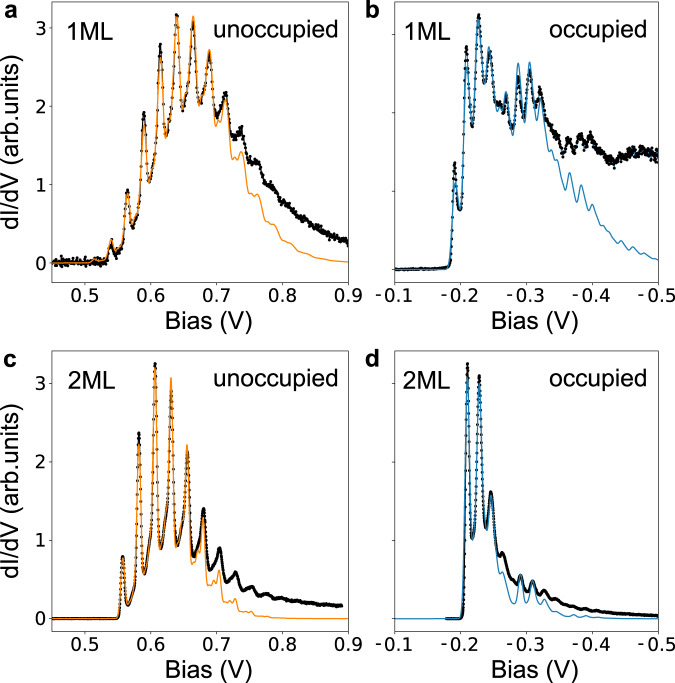
Vibronic excitations associated with charge state transitions of CRI. **a** Electron attachment to the unoccupied defect state for C_S_ on monolayer WS_2_. d*I*/d*V* measurement (black dots) and three-mode electron–phonon coupling model (orange line). **b** Hole attachment to the occupied defect state for C_S_ on monolayer WS_2_. d*I*/d*V* measurement (black dots) and four-mode electron–phonon coupling model (blue line). **c** Electron attachment for C_S_ on bilayer WS_2_. d*I*/d*V* measurement (black dots) and three-mode electron–phonon coupling model (orange line). **d** Hole attachment for C_S_ on bilayer WS_2_. d*I*/d*V* measurement (black dots) and four-mode electron–phonon coupling model (blue line). Fit parameters are given in Table 1.

From this analysis, we conclude the difference in vibrational coupling strength for the two defect states results from a combination of spin-dependent exchange and the distinct defect wavefunction character of the two states. These results are summarized in Fig. 5. As will be discussed shortly, the difference in coupling strengths is what ultimately gives rise to the very different sideband structure shown in Fig. 6a, b.

Repeating our calculations for a CRI defect in a WS_2_ bilayer identifies the same modes with significant coupling strengths as for monolayer, but with generally smaller *S* values (see Fig. 5a, b). This indicates that the defect states change less as the local vibration is excited in the bilayer system. In the bilayer, the defect states are delocalized into both layers, as shown in Supplementary Fig. 14, reducing the coupling to the lattice vibration. For the monolayer, our calculations also reveal strong coupling to low-frequency resonant flexural modes (Fig. 5c) that involve the CRI defect (*ℏω* ≈ 5 meV), particularly for the occupied CRI state. We note that a hybrid acoustic-CRI defect vibrational mode is more sensitive to supercell size, increasing the uncertainty of our calculated *S* values as detailed in the Supplementary Materials.

While tempting to use the DFT values for *ω*_*ν*_ and *S*_*νσ*_ in conjunction with Eq. (5) and compare directly with experimental STS data, we find that in practice *A*_*σ*_(*ω*) is quite sensitive to small numerical uncertainties in the Huang–Rhys factors introduced by our approximate calculations, e.g., choice of XC functional. Instead, guided by the small number of phonon modes with significant coupling strength identified by theory, we fit the measured STS spectra using Eq. (5) and subsequently compare the fit parameters to those obtained from DFT. For the unoccupied CRI state (electron attachment), three modes were sufficient for a good fit to the data; while for the occupied CRI state (hole attachment), four modes were used to largely reproduce all vibronic peaks. The fits to the tunneling spectra are shown in Fig. 6, while the refined values for *ω*_*ν*_ and *S*_*νσ*_ used in these fits are reported in Table 1. All vibronic resonances exhibit a homogeneous Gaussian line broadening (*σ* = 3–4 meV). Hence, this broadening is likely not temperature or lifetime limited but may be induced by coupling of the local vibrations to lattice acoustic modes^52^.

**Table 1:** Layer and charge state dependence of the CRI vibronic coupling in WS_2_.

	Unoccupied		Occupied	
	1 ML	2 ML	1 ML	2 ML
ℏ*ω*_1_ (meV)	10	10	6	10
*S*_1_	0.6	0.2	0.6	0.3
ℏ*ω*_2_ (meV)	19	16	**17**	**18**
*S*_2_	0.7	0.4	**2.2**	**0.9**
ℏ*ω*_3_ (meV)	**25**	**24**	26	23
*S*_3_	**5.4**	**2.8**	0.4	0.2
ℏ*ω*_4_ (meV)	–	–	79	81
*S*_4_	–	–	0.7	0.2
*σ* (meV)	4	3	4	3
$\bar{\epsilon }$ (meV)	515	560	−190	−210

Fitted Huang–Rhys factor *S*, vibrational energy ℏ*ω*, Gaussian broadening *σ*, and the electronic defect state energy $\bar{\epsilon }$ of the three-mode (unoccupied state) and four-mode (occupied state) electron-phonon model shown in Fig. 6. The mode with the highest coupling strength is printed in bold.

We pause to note that the values reported in Table 1 are not entirely unique in that different sets of *ω*_*ν*_ and *S*_*νσ*_ may be able to reproduce the STS data equally well. Thus care must be taken in interpreting the results. Nevertheless, there are some robust features independent of how the fit is performed which we can confidently compare with the DFT frequencies and couplings. Notably, we find that the pristine Frank–Condon-like sideband structure observed for the unoccupied defect state (Fig. 6a) derives primarily from strong defect coupling (*S* ≈ 5) to a single low frequency (*ℏω* ≈ 20 meV) vibrational mode. Conversely, the more complex sideband structure seen in the occupied defect-state STS spectra (Fig. 6b) primarily originates from two modes, a low-frequency mode (*ℏω* ≈ 20 meV) with moderate coupling (*S* ≈ 2) and a high-frequency mode (*ℏω* ≈ 80 meV) with a weaker coupling (*S* ≈ 1), the latter manifesting a beating pattern in the STS data. Remarkably, our DFT results are consistent with these results.

In short, we identify two vibrational modes, involving the CRI defect, that couple strongly to its defect states. While all mode energies are similar for both states and layer independent, their coupling strength is greatest for monolayer WS_2_ and is sensitive to the CRI defect state, indicating strong spin-phonon coupling in this system.

In summary, we demonstrate the selective and atomically precise generation of CRIs (C${}_{\,{{\mbox{S}}}\,}^{\bullet -}$) in a TMD host crystal. This is achieved by atomic editing of the TMD surface via STM-induced hydrogen depassivation. In its anionic state, the CRI forms an effective spin-1/2 system in the bandgap with a calculated magnetic moment of 1 μ_B_. Complementary measurements would be of use to confirm the calculated spin character of CRI in a magnetic field, for instance using ESR-STM. Synthetically introduced CH impurities are depassivated by H desorption using an STM tip. The resulting dangling bond introduces a deep defect state in the TMD bandgap that can be populated by electrons from the graphene substrate. For WS_2_ on Gr/SiC, the Fermi level alignment leads to a negative charge state of the C_S_ impurity, resulting in an open-shell, spin-polarized in-gap state. We also demonstrate that the atomic defect couples predominantly to two vibronic modes. While the vibrational frequencies largely defect state and layer independent, we find that the electron–phonon coupling strength is stronger for monolayer WS_2_ as compared to bilayer WS_2_. The different coupling strengths to the spin-polarized defect states are a manifestation of the spin-dependent vibronic coupling in CRIs.

## Methods

### Sample preparation

Metalorganic chemical vapor deposition synthesis of monolayer tungsten diselenide (WSe_2_) on graphene on silicon carbide (SiC) was performed from tungsten hexacarbonyl [W(CO)_6_, 99.99%, Sigma-Aldrich] and hydrogen selenide (H_2_Se, 99.99%, Matheson) precursors in a hydrogen gas atmosphere as previously reported.^53^ Monolayer islands of tungsten disulfide (WS_2_) on graphene/SiC were grown on graphene/SiC substrates with an ambient pressure CVD approach from tungsten oxide (WO_3_) and sulfur powder under argon gas.^54,55^ An inductively coupled plasma-enhanced CVD system was used to carry out the post-growth CH doping under an Ar/H_2_ mixture and with 1 sccm methane CH_4_ flow.^17^ Raman spectroscopy was used to characterize the sample, as discussed previously.^16,17^ After ex-situ growth, samples were transferred to ultrahigh vacuum (<2 × 10^−10^ mbar) and annealed at 200 °C to remove adsorbates.

### Scanning probe microscopy (SPM) measurements

SPM measurements were acquired with a Createc GmbH scanning probe microscope at liquid helium temperatures (*T* < 7 K) under an ultrahigh vacuum (*p* < 2 × 10^−10^ mbar). The quartz crystal cantilever (qPlus based) sensor^56^ tip apex was prepared by indentations into a gold substrate and verified as metallic on the Au(111) surface. Non-contact AFM images were taken with a carbon monoxide functionalized tip^57^ in constant height mode at zero bias. STM topographic measurements were taken in constant current mode with the bias applied to the sample. STS measurements were recorded using a lock-in amplifier with a resonance frequency of 683 Hz and a modulation amplitude between 2 and 10 mV.

### Vibronic model

Our STS measurements of the defect states feature a characteristic sideband structure as shown in Fig. 6, arising from strong electron–phonon interactions^51,58^. In our experimental setup, resonant tunneling between the tip and sample will occur when the applied bias voltage is aligned with the defect energy levels. The defect-phonon system can be described by an effective independent spin-boson Hamiltonian^50^, that is

3 $$H={\epsilon }_{\sigma }{c}_{\sigma }^{{{{\dagger}}} }{c}_{\sigma }+\mathop{\sum}\limits_{\nu }{\omega }_{\nu }{a}_{\nu }^{{{{\dagger}}} }{a}_{\nu }+\mathop{\sum}\limits_{\nu }{g}_{\nu \sigma }{c}_{\sigma }^{{{{\dagger}}} }{c}_{\sigma }({a}_{\nu }+{a}_{\nu }^{{{{\dagger}}} }),$$

where *ϵ*_*σ*_ is the energy level of the defect with spin state *σ*, ${c}_{\sigma }({c}_{\sigma }^{{{{\dagger}}} })$ the defect annihilation (creation) operator, *ω*_*ν*_ phonon frequency of mode *ν*, and ${a}_{\nu }({a}_{\nu }^{{{{\dagger}}} })$ phonon annihilation (creation) operator, and *g*_*νσ*_ the defect-phonon coupling matrix element.

The Hamiltonian in Eq. (3) can be solved exactly via a canonical transformation to arrive at the *T* = 0 electronic Green’s function^50^

4 $${G}_{\sigma }(t)=-i{{\Theta }}(t){e}^{-it({\epsilon }_{\sigma }-{{{\Omega }}}_{\sigma })}{e}^{-{{{\Phi }}}_{\sigma }(t)},$$

where ${{{\Phi }}}_{\sigma }(t)={\sum }_{\nu }{S}_{\nu \sigma }(1-{e}^{i{\omega }_{\nu }t})$ is the phonon contribution, ${S}_{\nu \sigma }={({g}_{\nu \sigma }/{\omega }_{\nu })}^{2}$ the coupling constant (also known as Huang–Rhys factor), and Ω_*σ*_ = ∑_*ν*_*S*_*νσ*_*ω*_*ν*_ the self-energy. We can derive the defect energy level spectral function by taking the imaginary part of the retarded Green’s function, obtaining

5 $${A}_{\sigma }(\omega )=2\pi \mathop{\sum }\limits_{\{{l}_{1}{l}_{2}...{l}_{n}\}}^{\infty }\left[\left(\mathop{\prod }\limits_{\nu =1}^{n}{e}^{-{S}_{\nu \sigma }}\frac{{S}_{\nu \sigma }^{{l}_{\nu }}}{{l}_{\nu }!}\right)\delta \left(\hslash \omega -{\bar{\epsilon }}_{\sigma }-\mathop{\sum }\limits_{\mu =1}^{n}\hslash {\omega }_{\mu }{l}_{\mu }\right)\right],$$

where *n* denotes the number of the phonon modes that couple to the defect. In the main text, we define ${\bar{\epsilon }}_{\sigma }={\epsilon }_{\sigma }-{{{\Omega }}}_{\sigma }$ since the Ω_*σ*_ only gives a relative shift of the defect level, and is not responsible for the fine structure of the sidebands.

To more closely model the experimental set-up, we note that Wingreen et al.^51^ added the hopping terms coupling of the contact leads with the defect state to the Hamiltonian in Eq. (3) and derived an expression for the resonant tunneling transmission probability. They found that when the hopping rate between contact and defect is assumed constant, the aforementioned transmission probability is proportional to the spectral function reported above, justifying a direct comparison of spectral function (5) with the transmission probability and ultimately the experimentally measured dI/dV curves in the main text.

### First-principles calculations

In this work, the spectral function (5) is constructed in a completely ab initio manner with no adjustable parameters. We calculate *ϵ*_*σ*_, *ω*_*ν*_, and *S*_*νσ*_ from first principles, as follows. First, for a fully optimized supercell, we perform a spin-polarized density functional theory (DFT) calculation to determine the Eigen energy associated with the defect state, *ϵ*_*σ*_. Using the same supercell, we then calculate *ω*_*ν*_ at the Γ-point of the Brillouin zone using density-functional perturbation theory (DFPT)^59^. After obtaining *ω*_*ν*_ and the corresponding (mass reduced) eigenmodes ***ξ***^*ν*^, we construct modulated structures freezing in displacement patterns associated with all phonon modes using PHONOPY^60^. The defect-phonon coupling matrix elements are then calculated with the following the finite difference method

6 $${g}_{\nu \sigma }=\frac{1}{\sqrt{2{\omega }_{\nu }}}\mathop{\lim }\limits_{A\to 0}[{\epsilon }_{\sigma }({{{{{{{{\boldsymbol{R}}}}}}}}}_{0}+A{{{{{{{{\boldsymbol{\xi }}}}}}}}}^{\nu })-{\epsilon }_{\sigma }({{{{{{{{\boldsymbol{R}}}}}}}}}_{0})]/A,$$

where *ϵ*_*σ*_(***R***_0_) is the defect energy level with nuclei fixed at their equilibrium configuration, ***R***_0_, while *ϵ*_*σ*_(***R***_0_ + *A****ξ***^*ν*^) denotes the defect energy when nuclei are displaced, by amplitude *A*, along the phonon eigenmode ***ξ***^*ν*^. For each phonon mode, we then compute the change in energy of the defect levels as a function of amplitude, *A*, considering a few different amplitudes. We perform a linear fit of the defect energy level with amplitude, determining *g*_*νσ*_ from the slope [Eq. (6)]. We have explicitly confirmed, for non-spin polarized calculations, that this approach gives the same result (within 2 meV) as explicit extraction of the coupling from DFPT. In this analysis, we neglect SOC. Additional details regarding the first-principles calculation are provided below.

We performed first-principles DFT calculations using the QUANTUM ESPRESSO package.^61^ We employed the PBE generalized gradient approximation for XC functional^34^ and used scalar relativistic optimized norm-conserving Vanderbilt (ONCV) pseudopotentials for W, S, C, and H atoms from the PseudoDojo library^62^ in all cases unless explicitly including SOC within nonlinear DFT; in these cases, the fully relativistic pseudopotentials of the same type were used. We used an 80 Ry plane-wave cutoff energy. In order to model a charged system, we added one additional electron to the cell, along with compensating background charge. We used the experimental lattice parameter for WS_2_, 3.15 Å, throughout this work unless otherwise stated. We generated 3 × 3, 5 × 5, 6 × 6, and 7 × 7 supercells including a single point defect with a vacuum of ~16 Å. For our total energy (electron–phonon) calculations, we used Γ-centered uniform 4 × 4(3 × 3), 3 × 3(2 × 2), 2 × 2(2 × 2), and 2 × 2(1 × 1)*k*-grids for 3 × 3, 5 × 5, 6 × 6, and 7 × 7 supercells, respectively, with Marzari–Vanderbilt smearing with the spread of 0.001 Ry^63^. Our convergence thresholds for ionic minimization on forces and total energy are 10^−4^ and 10^−6^ in atomic units. We considered spin polarization, in a collinear framework, throughout our calculations.

As the phonon frequencies of 2D materials are quite sensitive with respect to lattice parameters, we used the experimental lattice parameter of 3.15 Å since the PBE optimized lattice parameter (3.19 Å) is larger than the experimental value, which results in lower phonon frequencies. Our convergence threshold for self-consistency in our DFPT calculations is set to 1.0 × 10^−18^ a.u.

## Supplementary information

Supplementary Information

## Data Availability

The datasets generated and/or analyzed during the current study are available from the corresponding author on reasonable request.
