# Supplementary material for: Spin-dependent vibronic response of a carbon radical ion in two-dimensional WS2
Source: Nat Commun. 2021 Dec 15;12:7287. doi: 10.1038/s41467-021-27585-x (PMC8674275; doi:10.1038/s41467-021-27585-x)
Supplement: Supplementary file 1 — Supplementary Information [file 41467_2021_27585_MOESM1_ESM.pdf]

Supplementary Information: Spin-dependent vibronic response of a carbon radical ion in two-dimensional WS_2

Katherine A. Cochrane,[1,*] Jun-Ho Lee*,[2,3] Christoph Kastl,[4] Jonah Haber,[2,3] Tianyi Zhang,[5,6] Azimkhan Kozhakhmetov,[5] Joshua A. Robinson,[5,6] Mauricio Terrones,[5,6,7] Jascha Repp,[8] Jeffrey B. Neaton,[2,3,9,†] Alexander Weber-Bargioni,[1,‡] and Bruno Schuler[1,10,§]

[1]*Molecular Foundry, Lawrence Berkeley National Laboratory, California 94720, USA*

[2]*Materials Sciences Division, Lawrence Berkeley National Laboratory, California 94720, USA*

[3]*Department of Physics, University of California at Berkeley, Berkeley, CA 94720, USA*

[4]*Walter-Schottky-Institut and Physik-Department, Technical University of Munich, Garching 85748, Germany*

[5]*Department of Materials Science and Engineering, The Pennsylvania State University, University Park, PA 16082*

[6]*Center for Two-Dimensional and Layered Materials, The Pennsylvania State University, University Park, PA, 16802*

[7]*Department of Physics and Department of Chemistry, The Pennsylvania State University, University Park, PA, 16802*

[8]*Institute of Experimental and Applied Physics, University of Regensburg, Regensburg 93040, Germany*

[9]*Kavli Energy Nanosciences Institute at Berkeley, Berkeley, CA 94720, USA*

[10]*nanotech@surfaces Laboratory, Empa – Swiss Federal Laboratories for Materials Science and Technology, Dübendorf 8600, Switzerland*

(Dated: November 22, 2021)

CONTENTS

Supplementary Experiments 3

Comparison of monolayer vs bilayer WS_2 3

Majority and minority conversion events 6

High resolution imaging of CH_S and C_S 7

STS of partially occupied $C_S^{\bullet-}$ orbitals 8

Fourier analysis of the vibronic spectrum 9

C_S in WSe_2 11

Supplementary Calculations 12

Electronic structure of the defect states 12

Evolution of the defect states 13

Electronic structure of the CRI in monolayer and bilayer WS_2 14

Origin of the distinct vibronic coupling of the spin-split defect states 15

Defect wavefunction in bilayer WS_2 18

Resonant low-energy phonon modes 18

Convergence test 19

Functional dependence of Huang-Rhys factor 21

Valley splitting 22

CO-tip nc-AFM simulations 22

References 22

SUPPLEMENTARY EXPERIMENTS

Comparison of monolayer vs bilayer WS_2

In this study, we examined carbon substituting chalcogen (C_X) defects in several transition metal dichalcogenides (TMDs), including monolayer (1ML) and bilayer (2ML) tungsten disulfide (WS_2). The majority of the sample coverage of the chemical vapor deposition (CVD) grown WS_2 was 1 or 2 monolayers, but small, multilayer terraces sometimes grew in the center of the ~ 20 micron TMD islands. An edge of such a multilayer area is observed in Supplementary Figure 1a. In this image, the lowest terrace is 1ML WS_2 and each step is an increase in 1ML up to 6ML. A step profile is shown in Supplementary Figure 1b.

When WS_2 is reduced to 1ML the previously indirect band gap becomes direct with band extrema at different points of the Brillouine zone. This leads to different features in scanning tunneling spectroscopy (STS) measurements, particularly the onset of the conduction and valence bands. It should be noted that the onset of the valence band of 1ML WS_2 can only be observed from close distances as the K states have a reduced tunnelling matrix and the spectrum is therefore dominated by the Γ states[1]. As is seen with WS_2,[1] a narrowing of the band gap is observed with increasing film thickness, Supplementary Figure 1c.

Scanning tunneling spectra along a line of C_S in 1ML and 2ML WS_2 are shown in Supplementary Figure 2a and b respectively. Besides the change in the electron-phonon coupling strength discussed in the main text, additional resonances are observed above the valence band edge that can be attributed to resonant and bound states of the Coulomb impurity[2] .Because the screening and contributions from the local band extrema are different, the binding energy and the character of these states vary between 1ML and 2ML as expected.

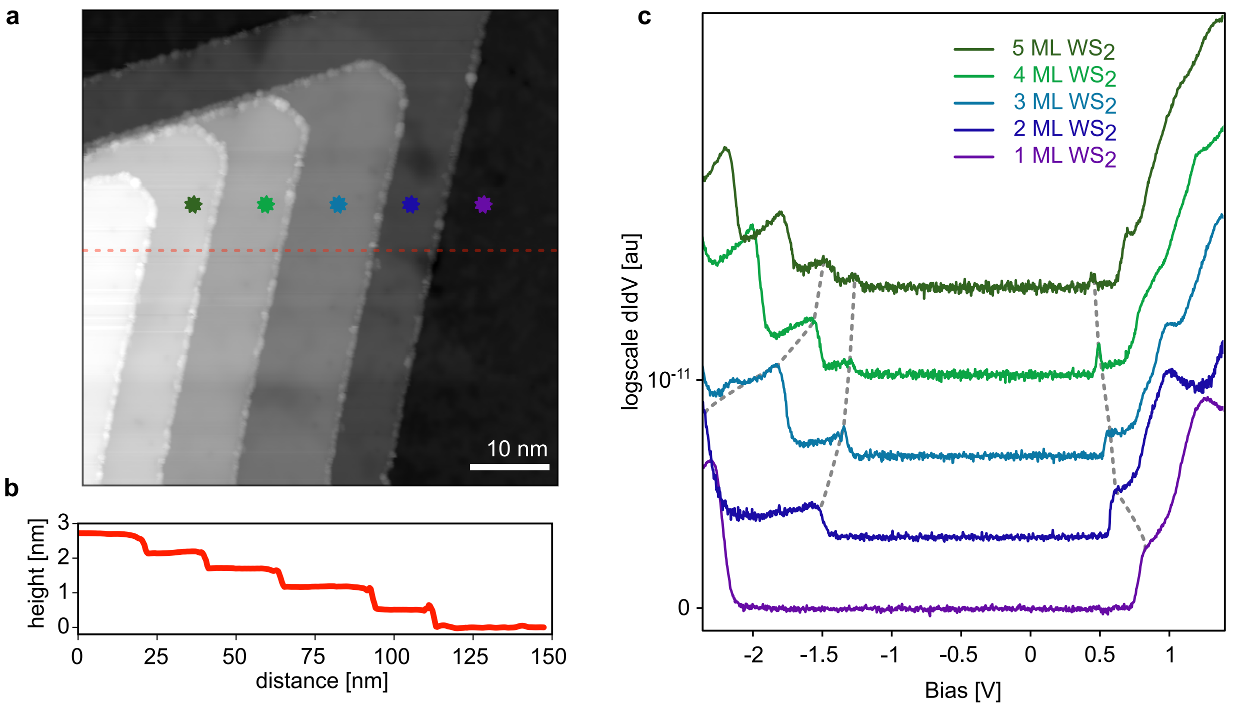

Supplementary Figure 1: **Layer-dependent scanning tunneling spectroscopy a**, STM of a WS_2 island showing terraces of 1 to 6 ML, $V_B = 1.5$ V, $I_t = 1$ nA, 150 x 150 nm. **b**, Height profile along the dashed red line indicated in (**a**). Average step height between terraces is approximately 0.5 nm. **c**, STS of 1 - 5 ML WS_2, setpoint V_B = 1.5 V, I_t = 1 nA, and V_{mod} = 10 mV.

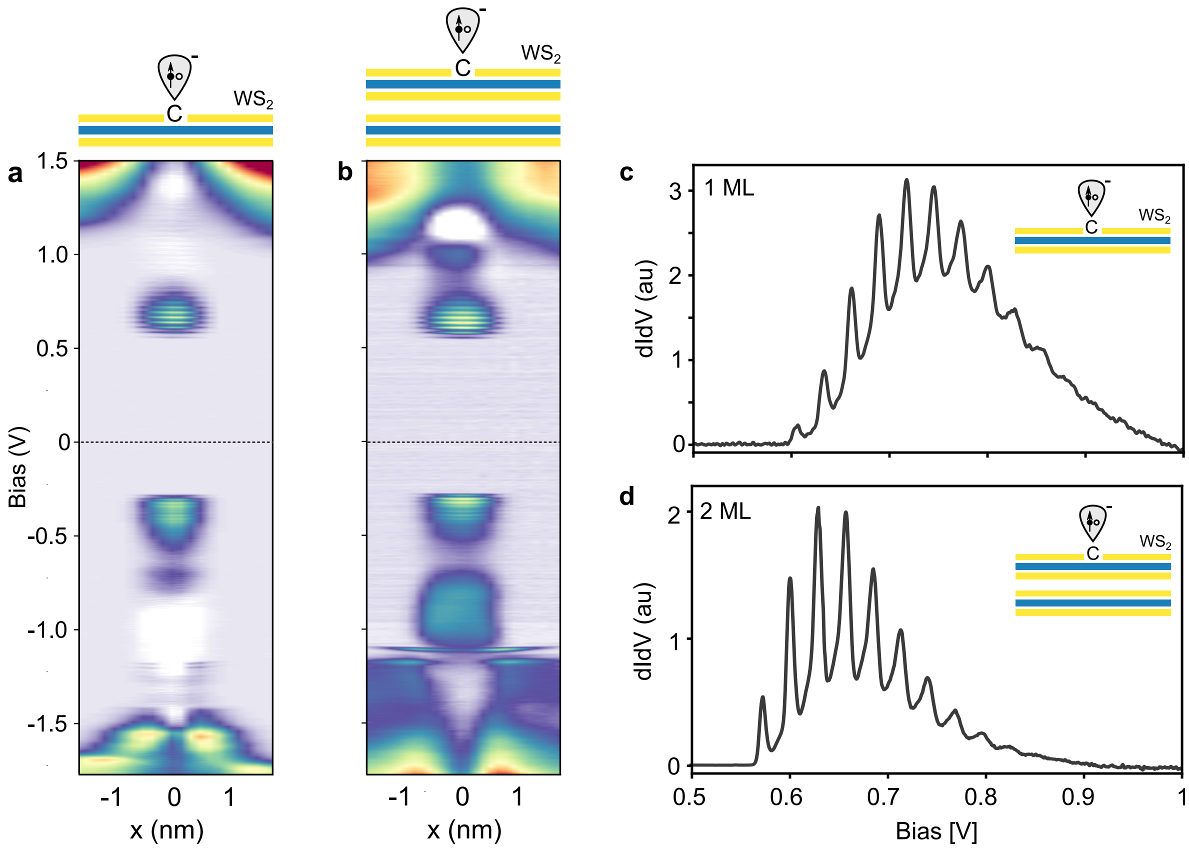

Supplementary Figure 2: **Comparison of tunneling spectra of a C_S defect on monolayer and bilayer WS_2 a,b**, Constant height point by point tunneling spectra acquired across the defect on monolayer (**a**) and bilayer (**b**) WS_2. Dotted black line indicates the Fermi energy. **c,d**, Point spectra of the converted defect on monolayer (**c**) and bilayer (**d**) WS_2.

Majority and minority conversion events

The dehydrogenation of CH_X reproducibly occurs at applied sample biases and tunneling currents above 2.5 V and 15 nA respectively. Supplementary Figure 3a shows a 20×20 nm^2 area of methane plasma treated, CVD grown WS_2 before any high currents or biases were applied; only CH_S and top and bottom O_S defects are observed. After a scan with dehydrogenation parameters, the majority of the time the hydrogen was removed from the carbon (Supplementary Figure 3b). However, occasionally the entire CH complex was removed and a sulfur vacancy was created, as indicated by the red circle in Supplementary Figure 3c. This creation of a sulfur vacancy was found to be an irreversible process. In addition, when passing over previously created C_S defects, occasionally reattachment of the hydrogen was seen as indicated by the yellow circle in Supplementary Figure 3d.

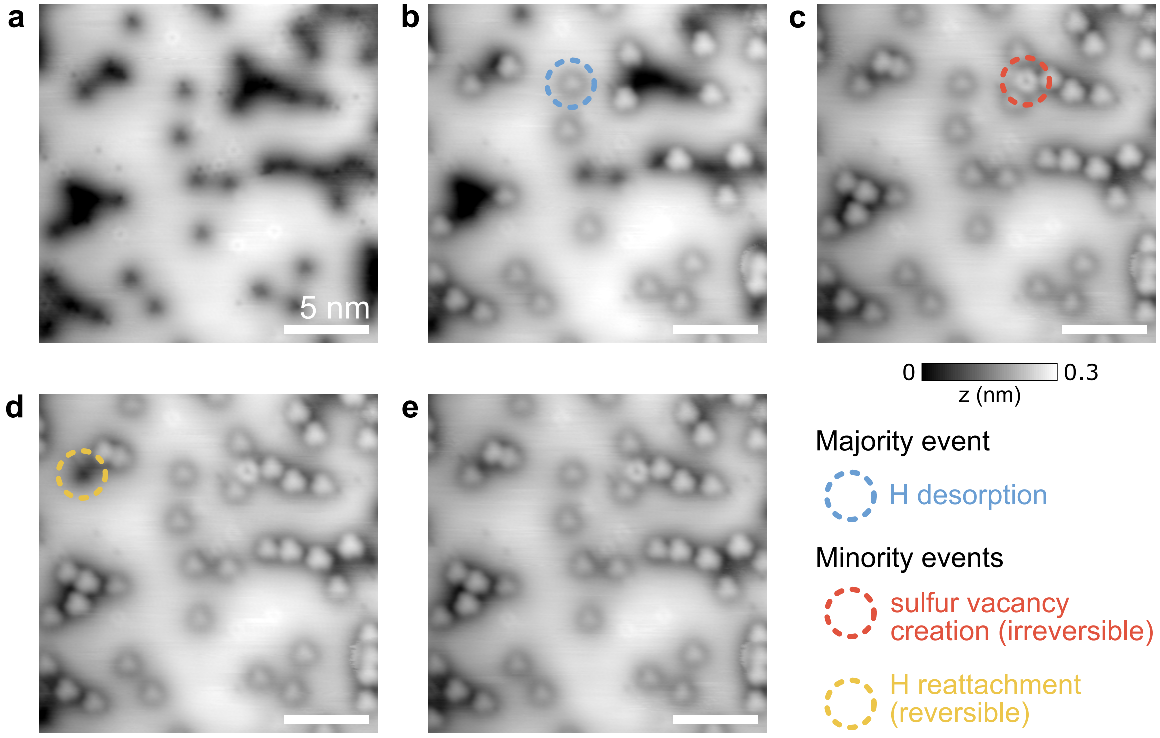

Supplementary Figure 3: **Sequential dehydrogenation with minority events a-e**, Sequential STM images taken after rastering the tip over CH_S defects while at dehydrogenation conditions of I_t =20 nA and V_B = 2.5 V. Scanning parameters: V_B = 1.2 V, I_t = 100 pA, 20×20 nm^2, sample was CVD grown and methane plasma doped WS_2.

High resolution imaging of CH_S and C_S

Supplementary Figure 4 shows a scanning tunneling microscopy overview image before and after the hydrogen removal process. After one pass with dehydrogenation parameters (biases and tunneling currents above 2.5 V and 15 nA respectively), a majority of the CH_S defects have been converted into C_S defects. Top and bottom oxygen at sulfur substitutions are also observed and remain unchanged, indicating the selectivity of the process.

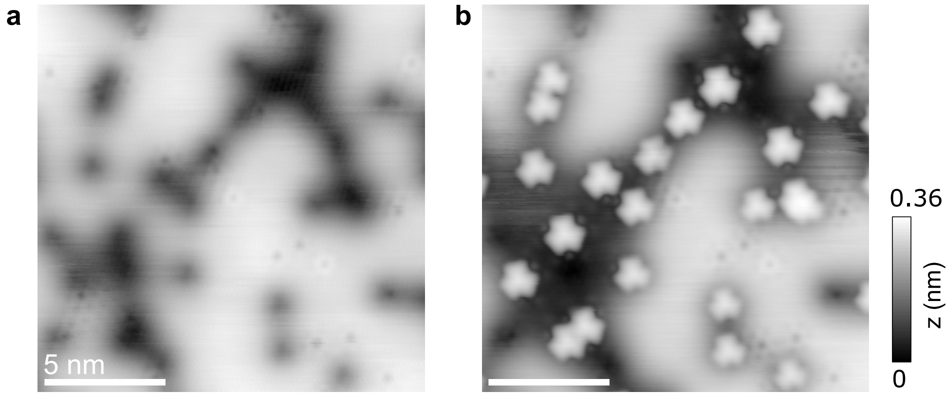

Supplementary Figure 4: **Selectivity of dehydrogenation process a,b**, STM image of CH doped WS_2 before (**a**) and after (**b**) dehydrogenation by high voltage and high currents. Scanning parameters: V_B = 1.1 V, I_t = 100 pA, 20 x20 nm.

Both the CH_S and the C_S defect are negatively charged, which results in the upwards bending of the substrate conduction and valence bands. Because of this, at positive biases these defects are imaged as dark depressions (in the case of CH_S, Supplementary Figure 5a) or with a dark depression surrounding the in-gap orbital features (C_S, Supplementary Figure 5b). If STM topographic images of the two defects taken under identical scanning parameters are subtracted from one another, the resulting image shows only the in-gap orbital from C_S, as seen in Supplementary Figure 5c. This indicates the degree of band banding, and therefore quantity of negative charge, is the same for both defects. The similar magnitude of band bending is also observed in the along a line tunneling spectra shown in the main paper (Figure 3a,b).

Supplementary Figure 5e shows a high resolution CO-functionalized tip AFM image of a side by side CH_S (left) and C_S (right) defect (corresponding STM of the same area is shown above

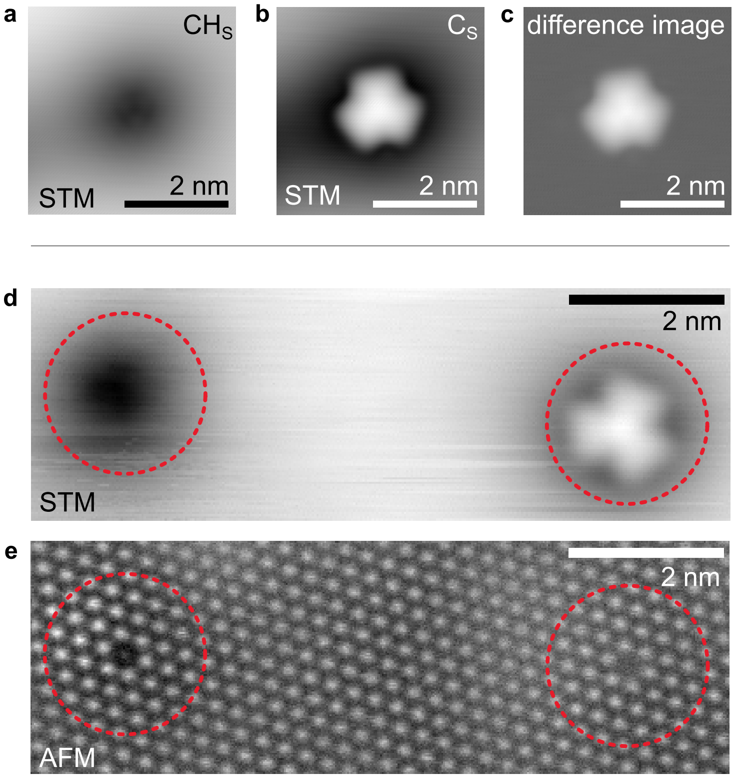

Supplementary Figure 5: **Comparison of CH_S and C_S atomic defect a,b**, STM constant current topographic image of a CH_S and a C_S defect respectively, $V_B = 1.1$ V, $I_t = 100$ pA, $4{\times}4$ nm^2. **c**, Difference image of (**a**) and (**b**) indicating the cancellation of the dark depression around the C_S defect, the component arising from the negative charge. **d**, STM and **e**, corresponding AFM images of side by side CH_S and a C_S defects, $9{\times}3$ nm^2. (STM: V_B = 0.73 V, I_t = 100 pA, AFM: V_B = 0 V).

in Supplementary Figure 5d). Just as in imaging of the isolated defects seen in the main text, the CH_S defect shows a subtle protrusion at the chalcogen site, while the C_S defect appears as a missing sulfur atom.

STS of partially occupied $C_S^{\bullet -}$ orbitals

The in gap resonances observed in the tunneling spectra of $C_S^{\bullet -}$, discussed in detail in the main text and shown again in Supplementary Figure 6a, arise from a singly occupied electronic orbital that is split into two spin-polarized states. Upon electron detachment at negative bias the defect state can be depopulated, whereas at positive bias an electron can be added to the

second spin-polarized state. The measured energetic splitting between the two states result from a combination of Coulomb repulsion and spin splitting.

Spatially resolved dI/dV maps were taken at five of the observed resonances, both above and below E_F. These maps appear spatially similar, indicating the resonances arises from the same electronic orbital.

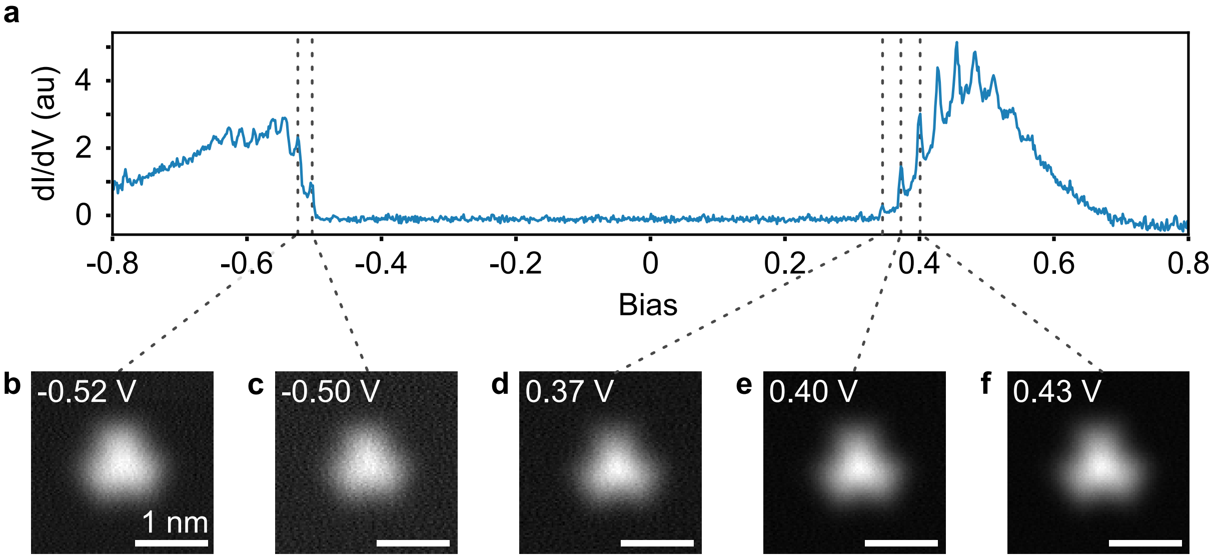

Supplementary Figure 6: **Scanning tunneling spectroscopy and orbital imaging of the CRI vibronic resonances a**, Scanning tunneling spectrum of a $C_S^{\bullet -}$ defect in 1ML WS_2 showing the occupied and unoccupied vibronic resonances. **b-f**, Spatially resolved STS maps of the singly occupied orbital of the C_S defect at negative (**b,c**) and positive (**d-f**) biases corresponding to resonances in the vibronic signal.

Fourier analysis of the vibronic spectrum

In the main text, the dI/dV spectra are fitted by Eq. (1). The lowest number of modes (and accordingly free fitting parameters) that are required to adequately describe the data are determined by an iterative process. Empirically we find that at least 3-modes are necessary to describe all features seen in the unoccupied defect spectrum, while a 4-mode model accounts for all spectral features in the occupied defect spectrum.

An alternative approach is to identify the dominant vibronic frequencies and their coupling strengths by a Fourier analysis. In Supplementary Figure 7 a Fourier analysis of the high-resolution dI/dV spectra shown in Figure 6 of the main text is depicted. The first column is the raw data, the second column the Fourier transform (FT), the third column the logarithm of the FT and the fourth column the fitted spectra using Eq. (1) with the main components identified in the FT (labelled in the second column).

While the Fourier transform reveals nice peaks for the unoccupied defect states with mode energies very similar to what we find by fitting the entire vibronic spectrum (Tab. 1 of the main text), the situation is less obvious for the occupied states. In particular, the back transformation does not fit the entire spectra equally well, as compared to the fitted spectra shown in Figure 6 of the main text. We assume that the limited number of periodic peaks (due to the small Huang-Rhys factor of some modes), limits the accuracy of the Fourier transformation. In the occupied dI/dV spectrum (negative sample bias) there might also be contributions from a non-constant tip density of states that would add some non-uniform background, which is a well-known effect in scanning tunneling spectroscopy. These factors complicate the Fourier analysis.

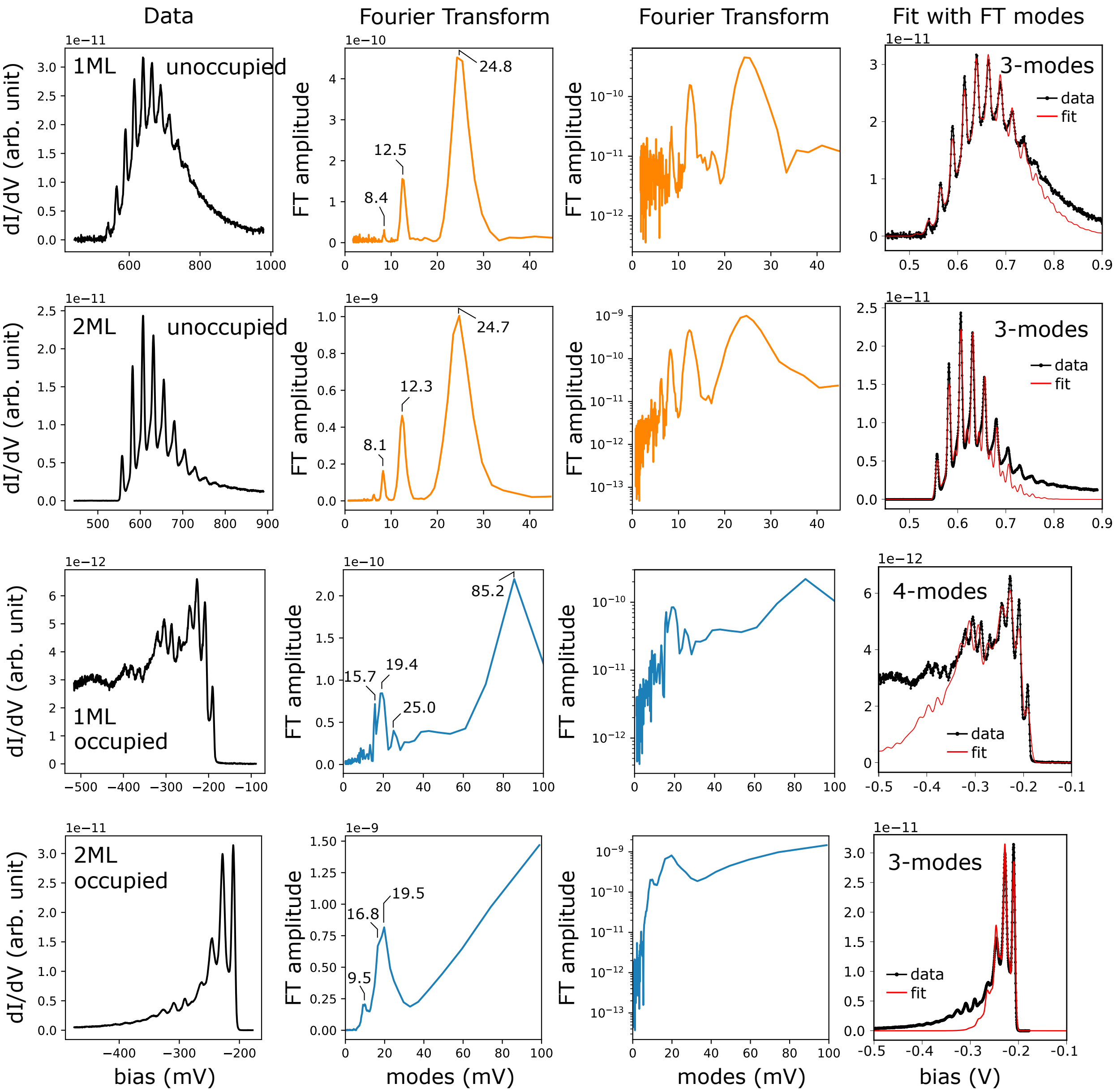

Supplementary Figure 7: **Fourier analysis of the vibronic spectra.** The Fourier components (second and third column) of the data shown in the first column. The main mode energies are labelled. The fourth column is a fit of the experimental data using Eq. (1) by fixing the frequencies $\hbar\omega_\mu$ to the values labelled in the second column. The fit deviates more from the data as compared to the values reported in Tab. 1 of the main text.

C_S in WSe_2

Experimental conditions for the dehydrogenation of CH_X for tungsten diselenide (WSe_2) are identical to those for WS_2, see Supplementary Figure 8. A significant difference between the two

materials is that even though both CH_S and CH_{Se} are both negatively charged, upon hydrogen removal, C_{Se} becomes neutral, while C_S remains negatively charged. This is seen in STM imaging by the absence of a dark depression surrounding the defect due to the lack of band bending, see Supplementary Figure 1b (in addition to the lack of splitting of the in gap resonance discussed in the main text).

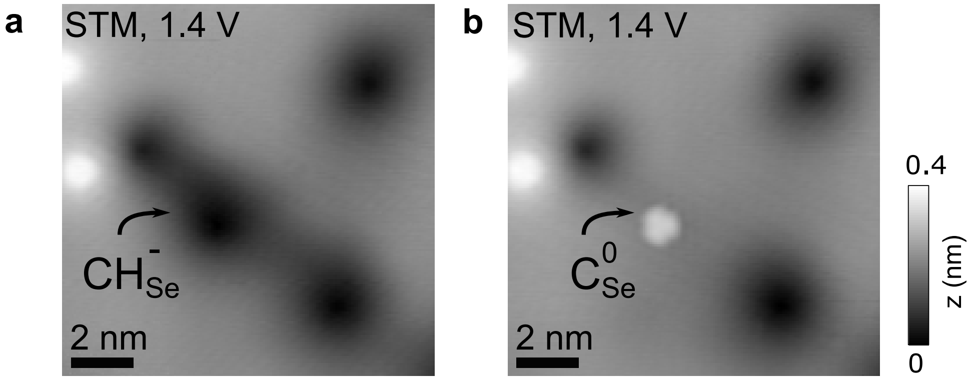

Supplementary Figure 8: **Hydrogen depassivation of C_{Se} in WSe_2.** STM constant current topographic image of an **a** CH_{Se} and a **b** C_{Se} defect respectively. Scanning parameters: $V_B = 1.5$ V, $I_t = 100$ pA, 15 x 15 nm.

SUPPLEMENTARY CALCULATIONS

Electronic structure of the defect states

In Supplementary Figure 9 we show our calculated density of states of the defect states with the integrated local density of states (ILDOS) that corresponds to the computed STM images. In particular, we project the defect wavefunction onto carbon atomic orbitals; carbon comprises about 33% of the defect state character. These states possess additional contributions from tungsten and sulfur states, indicating the defect states are hybridized (Supplementary Figure 11). ILDOS for the defect states (inset) closely resemble the experimental STM images. From our calculations, we find $m_j = +1/2$ ($m_j = -1/2$) for both $j = 3/2$ and $j = 1/2$ contribute to the occupied (unoccupied) state.

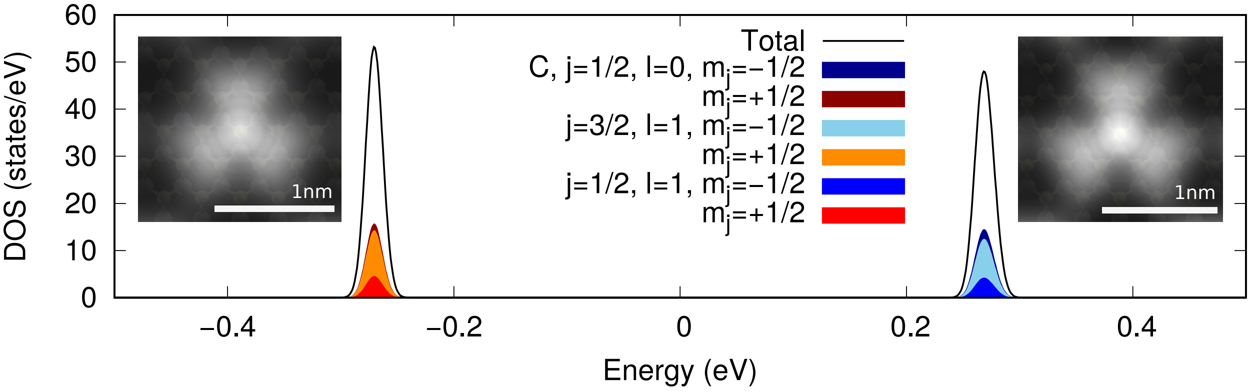

Supplementary Figure 9: **Projected density of CRI in-gap states.** Projected density of states and integrated local density of states of the defect states calculated by PBE with SOC in 5×5 supercell. Carbon contributions are shown. The centroid of the defect states is taken to be the zero of energy. Insets are integrated local density of states of the occupied and unoccupied defect states, respectively.

Evolution of the defect states

A carbon impurity substituting for a sulfur in monolayer WS_2 generates an unoccupied in-gap state as shown schematically in Supplementary Figure 10a [3,4]. Our WS_2 sample is placed on top of a graphene substrate. The relative Fermi level alignment of the WS_2/graphene system promotes charge transfer from graphene to monolayer WS_2 rendering the unoccupied defect state half-filled as shown in Supplementary Figure 10b. This half-filled metallic defect state is unstable against the spin-polarized state with two levels (Supplementary Figure 10c). Moreover, our vibronic analysis described above provides a complete explanation of the experimentally observed side-band features (Supplementary Figure 10d). Thus, we can rationalize the experimental observations by considering spin-polarization of the defect state with spin-dependent electron-phonon interaction. We anticipate that more rigorous experiments to measure magnetic properties of the impurity would consolidate our finding about the spin defect.

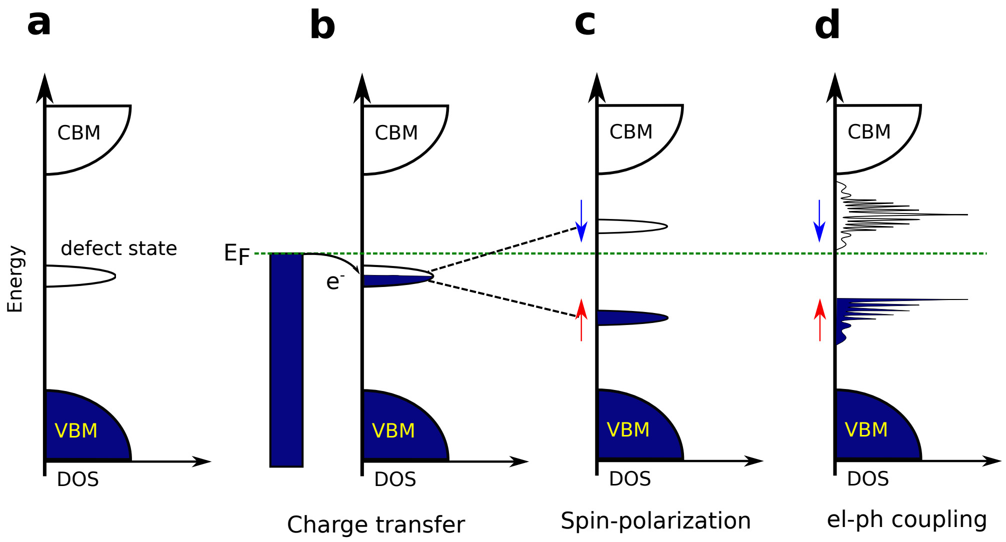

Supplementary Figure 10: **Defect state charging and spin polarization schematic.** Band structure of the charged carbon impurity in monolayer WS_2 with (a) nonmagnetic state and (b) spin-polarized state, respectively.

Electronic structure of the CRI in monolayer and bilayer WS_2

In Supplementary Figure 11 we show the projected density of state of the CRI in monolayer and bilayer WS_2, respectively. For our bilayer calculations, we used the LDA functional. The computed interlayer spacing, measured by difference in z-coordinate of W atom in each layer, is found to be 6.14 Å and the distance between top S in bottom WS_2 and bottom S in top WS_2 is found to be 3.04 Å. The defect level splitting is calculated to decrease in the bilayer.

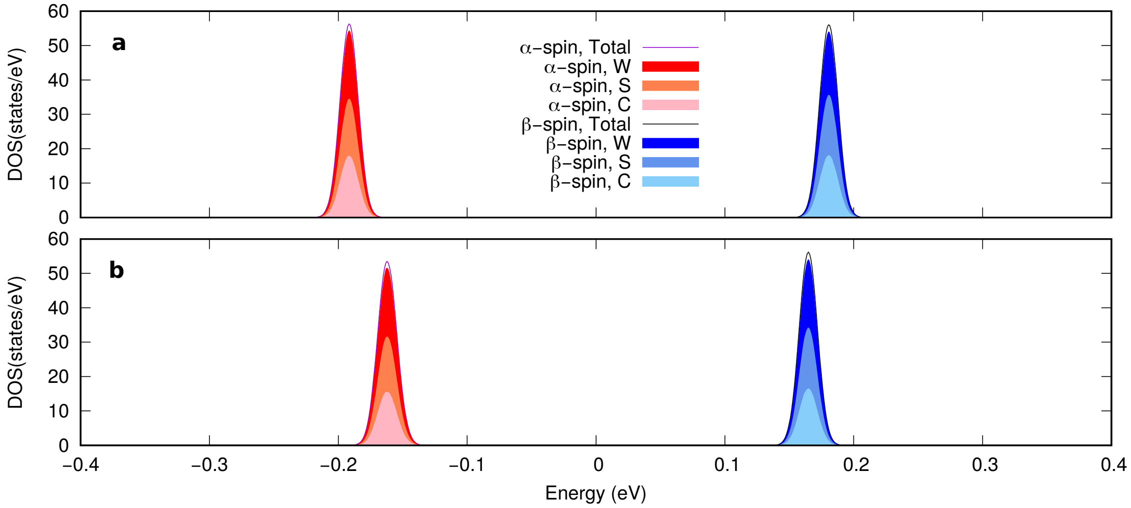

Supplementary Figure 11: **Monolayer vs bilayer projected density of CRI in-gap states**
a,b, Projected density of states of the CRI in (**a**) monolayer and (**b**) bilayer WS_2 with 6×6 supercell without SOC using LDA.

Origin of the distinct vibronic coupling of the spin-split defect states

The STM images of the spin-split defect states, at first glance, seem to be nearly identical, but there are meaningful differences between them that result in distinct electron-phonon coupling behavior for each defect state. Supplementary Figure 12 shows the difference of the density of each defect state ($\Delta\rho = \rho_{\text{occupied}} - \rho_{\text{unoccupied}}$), where $\rho_{\text{occupied}} = |\psi_{\text{occupied}}|^2$. The green isosurface stems from an excess electron density of the unoccupied state that is concentrated on the three neighboring W, C and bottom S atoms. The unoccupied defect state shows strong coupling with the 22 meV mode that is characterized by a breathing-like movement of these atoms. The red isosurface, resulting from an excess electron concentration of the occupied defect state, is mainly concentrated on the C atom. This state shows stronger coupling with the 75 meV mode that is characterized by an out-of-plane oscillation of the C atom. In this way, we see the trends we observe in $\Delta\rho$ are consistent the defect-phonon coupling matrix elements we compute from finite difference.

As detailed in the main text the exchange-correlation potential in the Kohn-Sham Hamiltonian may also give rise to some spin-dependent coupling. To analyze this, we partition the electron-

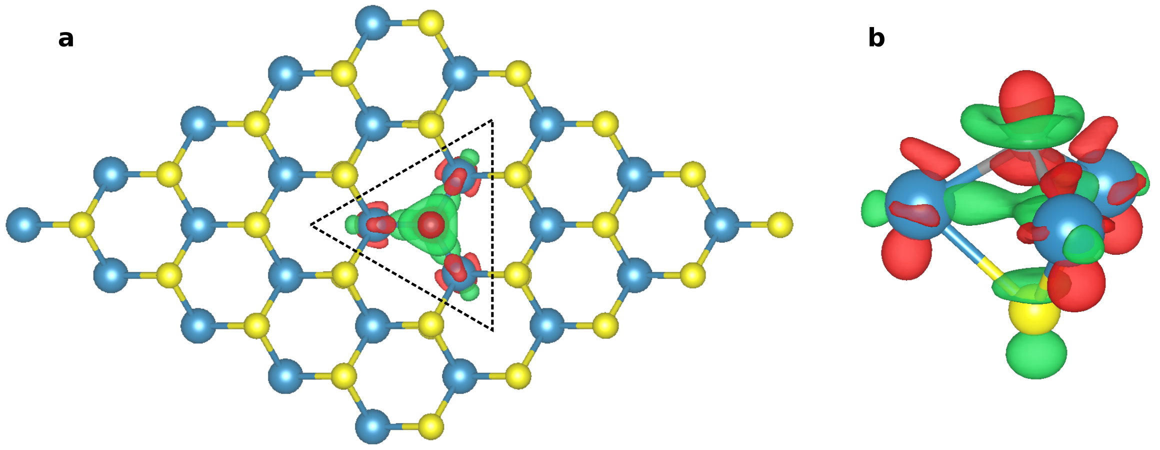

Supplementary Figure 12: **Difference in charge density a**, Top and **b**, perspective view of the difference of the defect states ($\Delta\rho$). The dashed triangle in (**a**) is highlighted in (**b**). Green (red) region has negative (positive) value and represents a distinct part that the unoccupied (occupied) defect state contributes. Isosurface is set to $\pm 0.8 \cdot 10^{-3} e/\text{Å}^3$

phonon matrix element as follows:

$$\begin{aligned} g_{\nu\sigma} &= \frac{1}{\sqrt{2\omega_\nu}} \langle \psi_\sigma | \partial_\nu H^{KS} | \psi_\sigma \rangle = \frac{1}{\sqrt{2\omega_\nu}} \langle \psi_\sigma | \partial_\nu (T + V_{\text{ext}} + V_{\text{H}}) | \psi_\sigma \rangle + \frac{1}{\sqrt{2\omega_\nu}} \langle \psi_\sigma | \partial_\nu V^\sigma_{\text{xc}} | \psi_\sigma \rangle \\ &= \frac{1}{\sqrt{2\omega_\nu}} \partial_\nu \langle \psi_\sigma | \, H^{KS} | \psi_\sigma \rangle = \frac{1}{\sqrt{2\omega_\nu}} \partial_\nu \langle \psi_\sigma | (T + V_{\text{ext}} + V_{\text{H}}) | \psi_\sigma \rangle + \frac{1}{\sqrt{2\omega_\nu}} \partial_\nu \langle \psi_\sigma | V^\sigma_{\text{xc}} | \psi_\sigma \rangle \end{aligned} \tag{1}$$

where the first term includes the kinetic (T), ionic (V_{ext}), and Hartree (V_{H}) potentials - all of which are spin-independent operators implying any difference in coupling between the two spin states, as far as this term is concerned, stems from the wavefunction ψ_σ. By contrast the second term involves the exchange-correlation potential V^σ_{xc}, an operator which does carry explicit spin dependence. Finally, the notation $\partial_\nu \mathcal{O}$ is a short hand meant to denote the change in some operator $\mathcal{O}$ when ionic degrees of freedom are displaced along phonon eigenmode $\boldsymbol{\xi}_\nu$. In the second line of 1 we use the Hellman-Feynman theorem to take the derivative outside the expectation value.

In practice, expectation values of these derivatives are evaluated through finite difference. For instance, for V^σ_{xc} we have

$$\partial_\nu \langle V^\sigma_{\text{xc}} \rangle \approx \lim_{A \to 0} [\langle \psi_\sigma(R_1) \, | V^\sigma_{\text{xc}}(R_1) | \, \psi_\sigma(R_1) \rangle - \langle \psi_\sigma(R_0) \, | V^\sigma_{\text{xc}}(R_0) | \, \psi_\sigma(R_0) \rangle] / A \tag{2}$$

where $R_1 = R_0 + A\boldsymbol{\xi}^\nu$ is a phonon-perturbed geometry by a phonon mode ν and R_0 is the ground state equilibrium geometry.

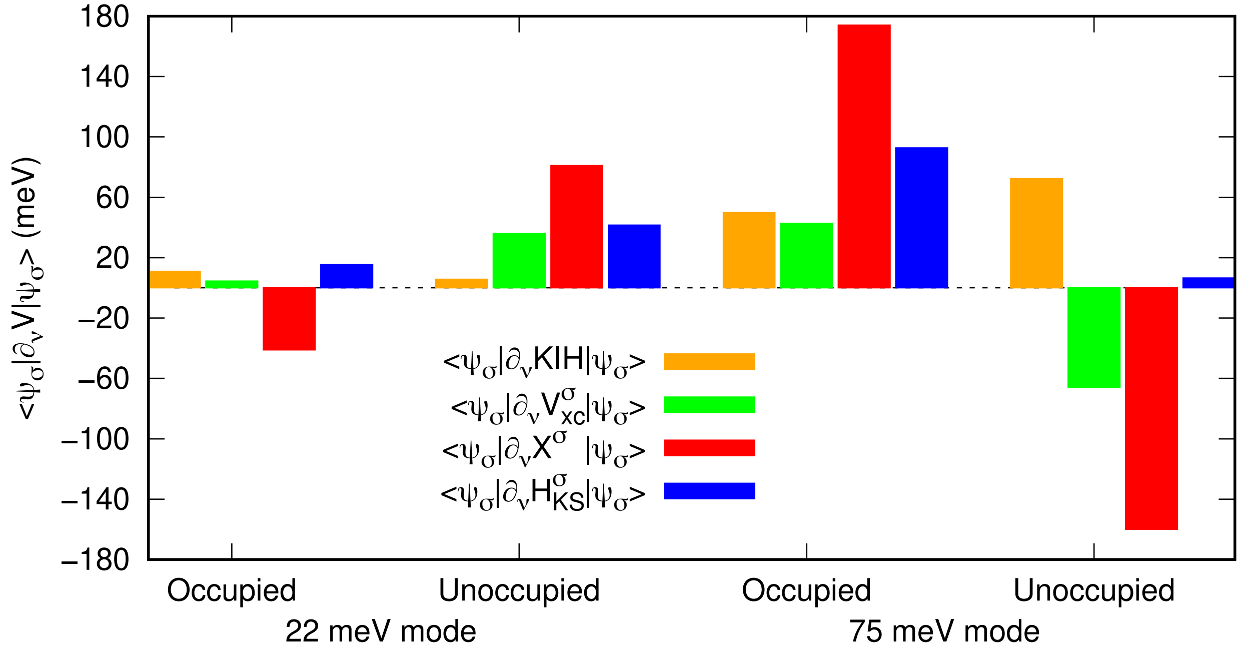

Supplementary Figure 13: **Energy decomposition of the CRI defect states.** Decomposition of the phonon-induced change of the matrix elements of Kohn-Sham (KS) Hamiltonian for the occupied ($\psi_\uparrow$) and unoccupied ($\psi_\downarrow$) defect states. Orange, green, red, and blue bars correspond kinetic, ionic, and Hartree terms (KIH), exchange-correlation, bare exchange, and KS Hamiltonian, respectively.

Supplementary Figure 13 shows the changes of the matrix elements upon phonon perturbation for each term. For the 22 meV mode that strongly couples to the *unoccupied* state, $\langle \partial_\nu V^\sigma_{\text{xc}} \rangle$ (green bar) displays the same trend as $\langle \partial_\nu H^\sigma_{\text{KS}} \rangle$, playing a decisive role in the large coupling strength. For the 75 meV mode on the other hand, where unoccupied state shows negligible coupling, $\langle V^\sigma_{\text{xc}} \rangle$ and contributions of the KIH term cancel each other out. We computed the bare Fock exchange contribution (red bars) as well to determine how robust this cancellation effect is to functional choice. Even with this drastic change in functional, we still find that the defect state which strongly couples to a 75 meV phonon mode has a positive contribution in the exchange part, while the weakly coupled defect state for the same mode has a negative contribution from the exchange part.

Defect wavefunction in bilayer WS_2

The defect wavefunction in bilayer WS_2 is not only localized in the layer that contains the carbon impurity, but also spreads in part into the bottom layer, particularly out to the W atom directly below the carbon impurity as shown in Supplementary Figure 14. Interlayer interactions lead to a slight delocalization of the defect state wavefunction.

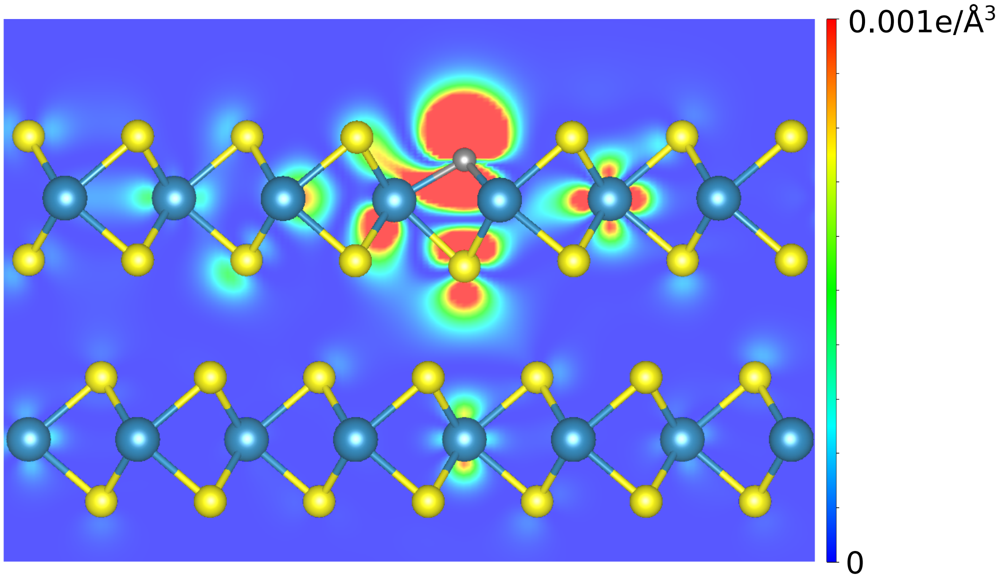

Supplementary Figure 14: **Defect state charge density in bilayer WS_2.** Defect wavefunction in bilayer WS_2 in a 2D plane that contains the carbon-tungsten bonds.

Resonant low-energy phonon modes

We can categorize two types of phonon modes that yield a finite coupling strength: local and resonant modes. Local phonon modes exhibit a flat dispersion and their energy and displacement patterns converge rapidly with increasing supercell size. Resonant, long wavelength phonons are more difficult to describe with our calculations as the choice of supercell and associated periodic boundary conditions impose a specific sampling of the reciprocal space. As we generate a supercell that is commensurate with the primitive WS_2 cell, some **q**-points of the primitive WS_2 cell are folded onto the Γ-point of the supercell. Thus, the Γ-point of each supercell contains *inequivalent* **q**-points that can be described as shown in Supplementary Figure 15, unless the size of the two supercells can be connected by integer multiplication. This means that by enlarging the size of the supercell we are not necessarily converging the resonant mode energy

and electron-phonon coupling; we are merely computing the electron-phonon coupling at the different **q**-points. In order to describe the interaction between an isolated point defect energy level and a phonon bath, considering Γ-point phonons is sufficient, as we wish to minimize artificial interactions between periodic images of defects in neighboring supercells.

As mentioned in the main text, our calculations have identified that the resonant mode that manifests strong coupling with the occupied defect states is a so called flexural phonon mode with an out of plane amplitude. In our supercell calculation, the flexural phonon in the 6×6 supercell is unique, leading to larger S than for 5×5 and 7×7 supercells. There are flexural modes in the 5×5 and 7×7 supercells as well, but the coupling strength is much smaller than that in the 6×6 supercell.

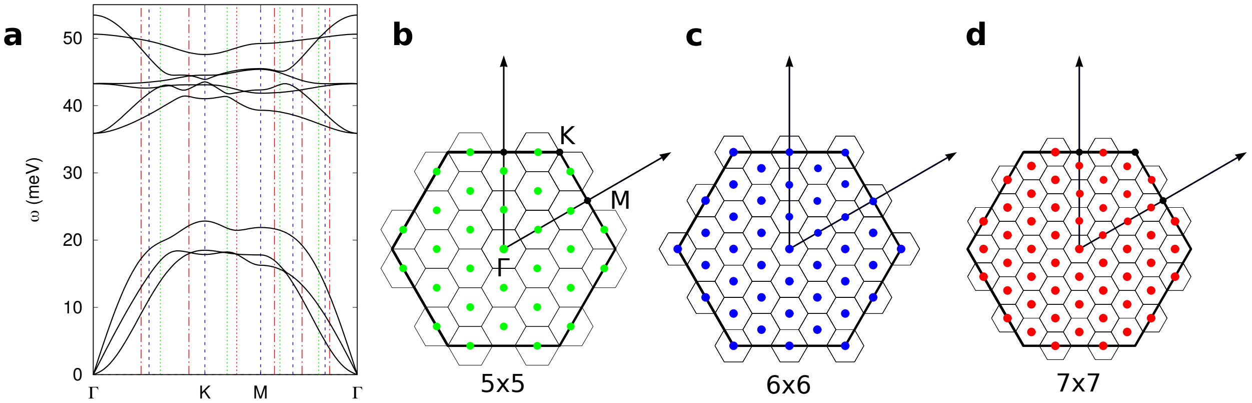

Supplementary Figure 15: **Phonon dispersion a**, Phonon dispersion of the monolayer WS_2 with the vertical lines that correspond **q**-points that can be expressed in the supercells. **b-d**, Brillouin zone of (**b**) 5×5, (**c**) 6×6, and (**d**) 7×7 supercells in that of the 1×1 unit.

Convergence test

We performed convergence tests for the calculated energy gain by spin-polarization and Huang-Rhys factor as a function of the supercell size as follows. We first calculated the energy difference between nonmagnetic and spin-polarized ground state as a function of supercell size, shown in Supplementary Figure 16, that is

$$\Delta E_{\text{sp}-\text{nm}} = E_{\text{sp}} - E_{\text{nm}}. \tag{3}$$

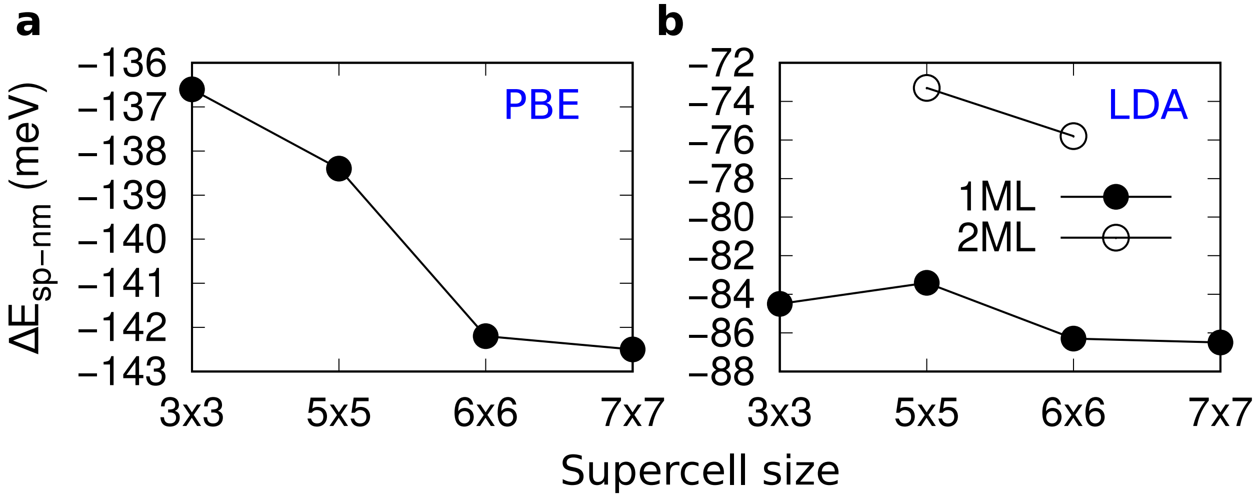

Supplementary Figure 16: **Convergence test a,b**, Calculated energy difference between the nonmagnetic and spin-polarized ground states with respect to supercell size using (**a**) PBE and (**b**) LDA functional, respectively. Filled (open) circles represent monolayer (bilayer) results.

We calculated $\Delta E_{\mathrm{sp-nm}}$ using PBE as a function of the supercell size as shown in Supplementary Figure 16a. $\Delta E_{\mathrm{sp-nm}}$ converges within 6 meV at the 6×6 supercell, which means the 6×6 supercell is sufficient to minimize the interactions between defects. The distance between CRIs in the 6×6 is $\sim$ 18.9 Å, whereas in the 5×5, it is $\sim$ 15.8 Å. When we calculate $\Delta E_{\mathrm{sp-nm}}$ using LDA, energy gain by spin-polarization is decreased by $\sim$ 56 meV (see Supplementary Figure 16b). LDA results also tend to converge at the 6×6 supercell. In the bilayer, $\Delta E_{\mathrm{sp-nm}}$ is $\sim$ 10 meV smaller than in the monolayer.

We also calculated Huang-Rhys factor as a function of the supercell size for the local phonon modes as shown in Supplementary Figure 17. The calculated Huang-Rhys factor of the two local phonon modes converge at the 5×5 supercell.

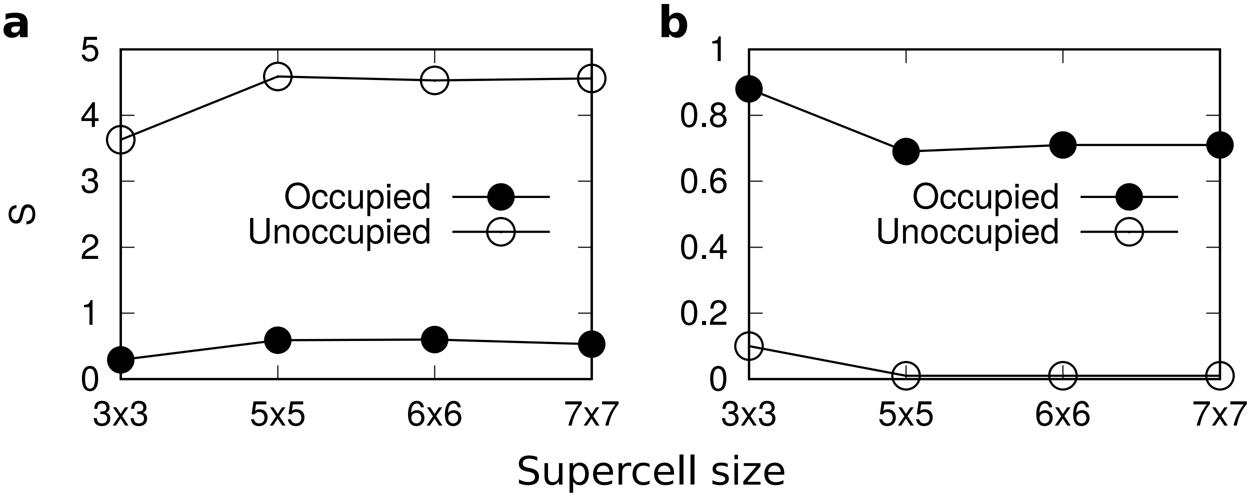

Supplementary Figure 17: **Calculated Huang-Rhys factors vs unit cell a,b**, Calculated Huang-Rhys factors for (**a**) 22 meV and (**b**) 75 meV local phonon mode as a function of supercell

Functional dependence of Huang-Rhys factor

We calculated the Huang-Rhys factor using a PBE and a LDA functional, respectively, as shown in Supplementary Figure 18. We find that PBE tends to result in larger S values than those using LDA, except for the coupling between the occupied and the 22 meV local phonon mode. The calculated Huang-Rhys factors in the bilayer are weaker than those in the monolayer.

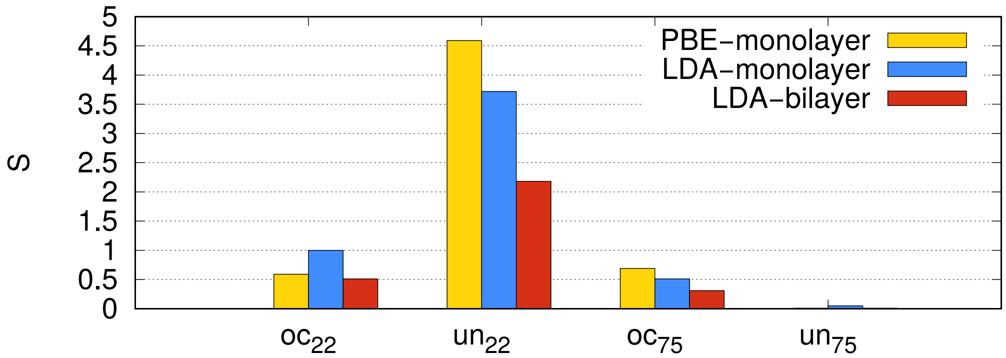

Supplementary Figure 18: **Calculated Huang-Rhys factors vs functional.** Calculated Huang-Rhys factor using PBE and LDA in a 5×5 supercell. oc_{22} (un_{75}) stands for coupling between the occupied (unoccupied) defect state and 22 (75) meV local phonon mode. Bilayer results calculated by LDA are shown.

Valley splitting

We calculated valley splitting induced by generation of CRI in monolayer WS_2, defined by

$$\lambda(\rho) = \epsilon_v^{\mathrm{K}}(\rho) - \epsilon_v^{\mathrm{K}'}(\rho) \tag{4}$$

where ϵ_v^{K} ($\epsilon_v^{\mathrm{K}'}$) is the energy of the valence band maximum at K (K′) valley, as a function of defect density, ρ, by changing supercell size, as shown in Supplementary Figure 19. For the sample defect densities, the valley splitting λ increases logarithmically as a function of magnetic defect density.

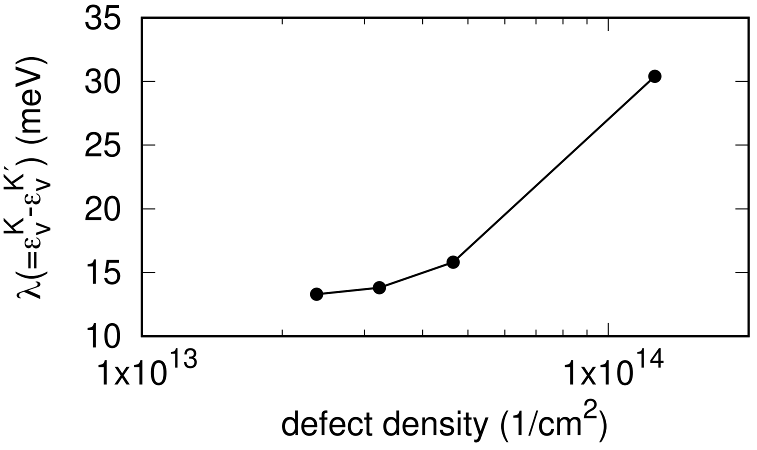

Supplementary Figure 19: **Calculated λ as a function of doping concentration.** The highest (lowest) defect density corresponds to a 3×3 and (7×7) supercell, respectively.

CO-tip nc-AFM simulations

Using the calculated relaxed unit cells for CH_S^- and C_S^-, we simulated CO-tip nc-AFM images using the probe particle model[5], shown in Supplementary Figure 20. For CH_S^-, the simulation resolves a small protrusion at the CH site that exhibits a weaker Δf contrast than the S sites, fully consistent with the measurement shown in Figure 2c. For C_S^-, there is no such protrusion at the defect site, also fully consistent with the AFM map after hydrogen removal (Figure 2d).

* These authors contributed equally to this work.

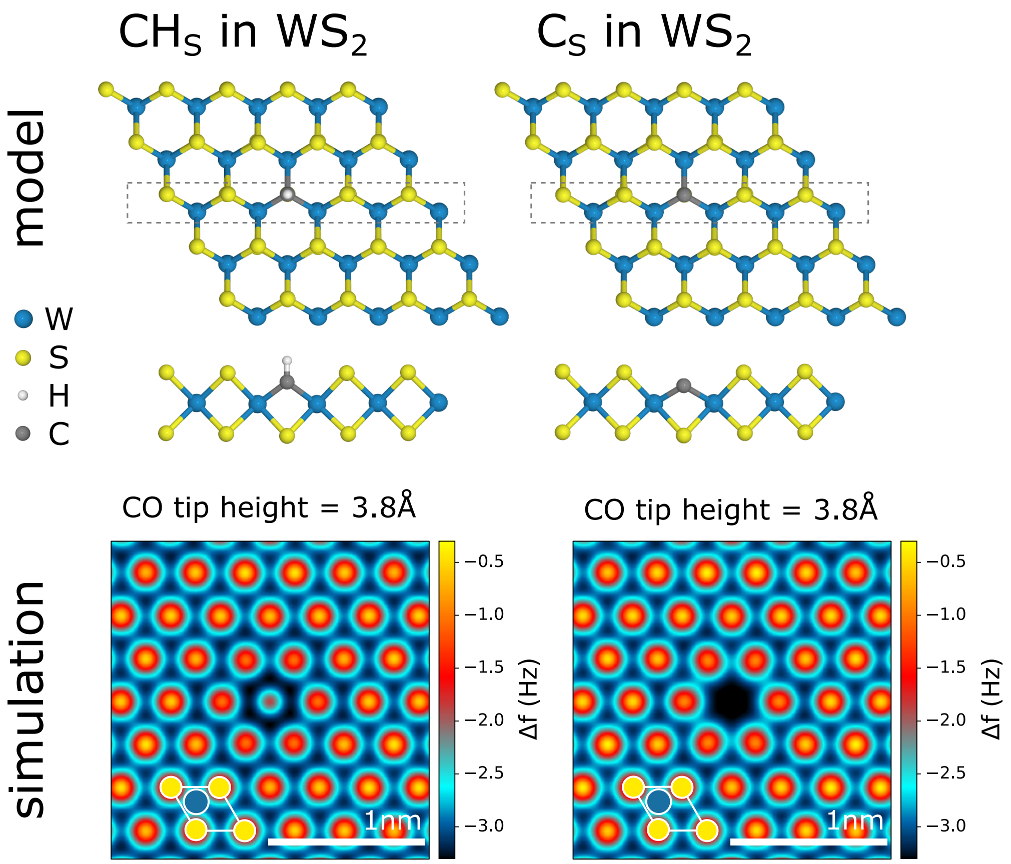

Supplementary Figure 20: **CO-tip nc-AFM simulation.** Atomic model and corresponding CO-tip nc-AFM simulation derived from the relaxed DFT geometry using the probe particle model by Hapala *et al.*[5]

† jbneaton@lbl.gov

‡ afweber-bargioni@lbl.gov

§ bruno.schuler@empa.ch

[1] Liu, H. *et al.* Molecular-beam epitaxy of monolayer and bilayer WSe_2: a scanning tunneling microscopy/spectroscopy study and deduction of exciton binding energy. *2D Materials* **2**, 034004 (2015).

[2] Aghajanian, M. *et al.* Resonant and bound states of charged defects in two-dimensional semiconductors. *Phys. Rev. B* **101**, 081201(R) (2020).

[3] Komsa, H. P. *et al.* Two-dimensional transition metal dichalcogenides under electron irradiation: Defect production and doping. *Phys. Rev. Lett.* **109**, 035503 (2012). 1206.4407.

[4] Zhang, F. *et al.* Carbon doping of WS_2 monolayers: Bandgap reduction and p-type doping transport. *Sci. Adv.* **5**, eaav5003 (2019).

[5] Hapala, P. *et al.* Mechanism of high-resolution stm/afm imaging with functionalized tips. *Phys. Rev.B* **90**, 085421 (2014).
